# dPeak: High Resolution Identification of Transcription Factor Binding Sites from PET and SET ChIP-Seq Data

**DOI:** 10.1371/journal.pcbi.1003246

**Published:** 2013-10-17

**Authors:** Dongjun Chung, Dan Park, Kevin Myers, Jeffrey Grass, Patricia Kiley, Robert Landick, Sündüz Keleş

**Affiliations:** 1 Department of Statistics, University of Wisconsin, Madison, Wisconsin, United States of America; 2 Department of Biomolecular Chemistry, University of Wisconsin, Madison, Wisconsin, United States of America; 3 Department of Biochemistry, University of Wisconsin, Madison, Wisconsin, United States of America; 4 Great Lakes Bioenergy Research Center, University of Wisconsin, Madison, Wisconsin, United States of America; 5 Department of Bacteriology, University of Wisconsin, Madison, Wisconsin, United States of America; 6 Department of Biostatistics and Medical Informatics, University of Wisconsin, Madison, Wisconsin, United States of America; Center for Genomic Regulation, Spain

## Abstract

Chromatin immunoprecipitation followed by high throughput sequencing (ChIP-Seq) has been successfully used for genome-wide profiling of transcription factor binding sites, histone modifications, and nucleosome occupancy in many model organisms and humans. Because the compact genomes of prokaryotes harbor many binding sites separated by only few base pairs, applications of ChIP-Seq in this domain have not reached their full potential. Applications in prokaryotic genomes are further hampered by the fact that well studied data analysis methods for ChIP-Seq do not result in a resolution required for deciphering the locations of nearby binding events. We generated single-end tag (SET) and paired-end tag (PET) ChIP-Seq data for 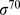 factor in *Escherichia coli (E. coli)*. Direct comparison of these datasets revealed that although PET assay enables higher resolution identification of binding events, standard ChIP-Seq analysis methods are not equipped to utilize PET-specific features of the data. To address this problem, we developed dPeak as a high resolution binding site identification (deconvolution) algorithm. dPeak implements a probabilistic model that accurately describes ChIP-Seq data generation process for both the SET and PET assays. For SET data, dPeak outperforms or performs comparably to the state-of-the-art high-resolution ChIP-Seq peak deconvolution algorithms such as PICS, GPS, and GEM. When coupled with PET data, dPeak significantly outperforms SET-based analysis with any of the current state-of-the-art methods. Experimental validations of a subset of dPeak predictions from 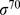 PET ChIP-Seq data indicate that dPeak can estimate locations of binding events with as high as 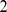 to 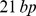 resolution. Applications of dPeak to 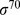 ChIP-Seq data in *E. coli* under aerobic and anaerobic conditions reveal closely located promoters that are differentially occupied and further illustrate the importance of high resolution analysis of ChIP-Seq data.

## Author Summary

Chromatin immunoprecipitation followed by high throughput sequencing (ChIP-Seq) is widely used for studying *in vivo* protein-DNA interactions genome-wide. Current state-of-the-art ChIP-Seq protocols utilize single-end tag (SET) assay which only sequences  ends of DNA fragments in the library. Although paired-end tag (PET) sequencing is routinely used in other applications of next generation sequencing, it has not been much adapted to ChIP-Seq. We illustrate both experimentally and computationally that PET sequencing significantly improves the resolution of ChIP-Seq experiments and enables ChIP-Seq applications in compact genomes like *Escherichia coli (E. coli)*. To enable efficient identification using PET ChIP-Seq data, we develop dPeak as a high resolution binding site identification algorithm. dPeak implements probabilistic models for both SET and PET data and facilitates efficient analysis of both data types. Applications of dPeak to deeply sequenced *E. coli* PET and SET ChIP-Seq data establish significantly better resolution of PET compared to SET sequencing.

## Introduction

**Figure 1:**
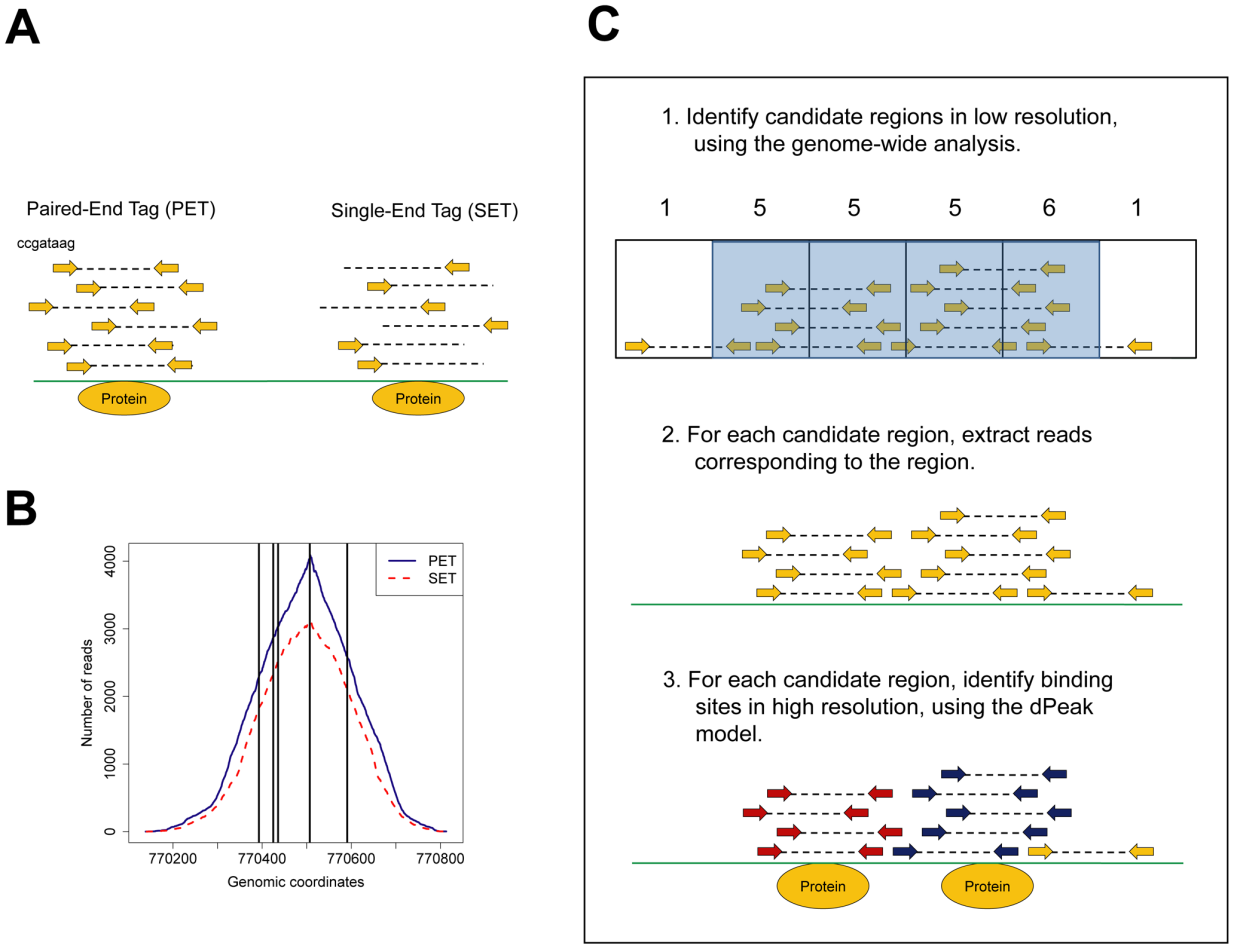
SET and PET ChIP-Seq data structure and the dPeak algorithm. (A) Description of paired-end tag (PET) and single-end tag (SET) ChIP-Seq data. Directions of arrows indicate strands of reads. (B) Promoter region of the *cydA* gene contains five closely spaced 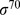 binding sites. Blue solid and red dotted curves indicate the number of extended reads mapping to each genomic coordinate in 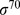 PET and SET ChIP-Seq data, respectively. Black vertical lines mark 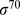 binding sites annotated in the RegulonDB database. (C) Pictorial depiction of the dPeak algorithm.

Since its introduction, chromatin immunoprecipitation followed by high throughput sequencing (ChIP-Seq) has revolutionized the study of gene regulation. ChIP-Seq is currently the state-of-the-art method for studying protein-DNA interactions genome-wide and is widely used [1]–[5]. ChIP-Seq experiments capture millions of *DNA fragments* (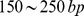 in length) that the protein under study interacts with using random fragmentation of DNA and a protein-specific antibody. Then, high throughput sequencing of a small region (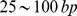) at the 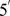 end or both ends of each fragment generates millions of *reads* or *tags*. Sequencing one end and both ends are referred to as *single-end tag (SET)* and *paired-end tag (PET)* technologies, respectively (Figure 1A). Standard preprocessing of these data involves mapping reads to a reference genome and retaining the uniquely mapping ones [6], [7]. In PET data, start and end positions of each DNA fragment can be obtained by connecting positions of paired reads [8]. In contrast, the location of only the 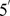 end of each DNA fragment is known in SET data. The usual practice for SET data is to either extend each read to its 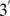 direction by the average library size which is a parameter set in the experimental procedure [7] or shift the 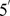 end position of each read by an estimate of the library size [9]. Then, genomic regions with large numbers of clustered aligned reads are identified as binding sites using one or more of the many available statistical approaches [6], [7], [9]–[11] (the first step in Figure 1C).

Currently, the SET assay dominates all the ChIP-Seq experiments despite the fact that PET has several obvious, albeit less studied, advantages over SET. In PET data, paired reads from both ends of each DNA fragment can reduce the alignment ambiguity, increase precision in assigning the fragment locations, and improve mapping rates. This is especially advantageous for studying regulatory roles of repetitive regions of genomes [12], [13]. Although many eukaryotic genomes are rich in repetitive elements, PET technology has not been extensively used with eukaryotic genomes [8], [14]. One of the main reasons for this is that ChIP-Seq data is information rich even when the repetitive regions are not profiled [15] and that the PET assay costs 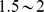 times more than the SET assay. Put differently, given a fixed cost, PET sequencing results in a lower sequencing depth compared to SET sequencing.

In contrast to eukaryotic genomes, prokaryotic genomes are highly mappable, e.g., 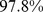 of the *Escherichia coli* (*E. coli*) genome is mappable with 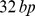 reads. This decreases the higher mapping rate appeal of the PET assay for these genomes. In this paper, we systematically investigate advantages of the PET assay from a new perspective and demonstrate both experimentally and computationally that it significantly improves the resolution of protein binding site identification. Improving resolution in identifying protein-DNA interaction sites is a critical issue in the study of prokaryotic genomes because prokaryotic transcription factors have closely spaced binding sites, some of which are only 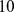 to 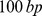 apart from each other [16]–[19]. These closely spaced binding sites are considered to be multiple “switches” that differentially regulate gene expression under diverse growth conditions [17]. Therefore, identification and differentiation of closely spaced binding sites are invaluable for elucidating the transcriptional networks of prokaryotic genomes.

Although many methods have been proposed to identify peaks from ChIP-Seq data (reviewed in [20]), such as MACS [9], CisGenome [6], and MOSAiCS [10], these approaches reveal protein binding sites only in low resolution, i.e., at an interval of hundreds to thousands of base pairs. Furthermore, they report only one “mode” or “predicted binding location” per peak. More recently, deconvolution algorithms such as CSDeconv [21], GPS [22] (recently improved as GEM [23]), and PICS [11] have been proposed to identify binding sites in higher resolution. However, these methods are specific to SET ChIP-Seq data and are not equipped to utilize the main features of PET ChIP-Seq data. Although a relatively recent method named SIPeS [24] is specifically designed for PET data and is shown to perform better than MACS paired-end mode [9], our extensive computational and experimental analysis indicated that this approach is not suited for identifying closely located binding events. To address these limitations, we developed dPeak, a high resolution binding site identification (deconvolution) algorithm that can utilize both PET and SET ChIP-Seq data. The dPeak algorithm implements a probabilistic model that accurately describes the ChIP-Seq data generation process and analytically quantifies the differences in resolution between the PET and SET ChIP-Seq assays. We demonstrate that dPeak outperforms or performs competitively with the available SET-specific methods such as PICS, GPS, and GEM. More importantly, dPeak coupled with PET ChIP-Seq data improves the resolution of binding site identification significantly compared to SET-based analysis with any of the available methods. Generation and analysis of 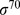 factor PET and SET ChIP-Seq data from *E. coli* grown under aerobic and anaerobic conditions reveal the power of the dPeak algorithm in identifying closely located binding sites. Our study demonstrates the importance of high resolution binding site identification when studying the same factor under diverse biological conditions. We further support our findings by validating a small subset of our closely located binding site predictions with primer extension experiments.

## Results

### Deeply sequenced *E. coli* 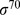 SET and PET ChIP-Seq data

The 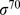 factor is responsible for transcription initiation at over 80% of the known promoters in *E. coli* [25]. 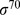 combines with RNA polymerase to bind promoter sequences typically containing two consensus elements located at 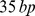 and 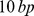 upstream of the transcription start site [18]; thus a 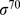 binding site spans about 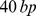 upstream from the transcription start site. Many *E. coli* genes contain multiple 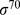 promoters, and much transcriptional regulation by oxygen as well as by other stimuli occurs by selection of one or a subset of the possible promoters in concert with binding of activators and repressors (e.g., ArcA and FNR for regulation by oxygen [17], [19]). Understanding such regulation requires knowledge of precisely which promoters are used in a given condition. Therefore, the highest possible accuracy of ChIP-signal mapping will allow the best determination of promoter binding by 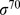-RNA polymerase holoenzyme.

We generated both PET and SET ChIP-Seq data for 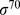 factor from *E. coli* grown under aerobic (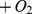) and anaerobic (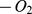) conditions in glucose minimal media on the HiSeq2000 and Illumina GA IIx platforms. We used these experimental data for comparisons of PET and SET assays and evaluation of our high resolution binding site detection method dPeak throughout the paper. Figure 1B displays PET and SET ChIP-Seq coverage plots for the promoter region of the *cydA* gene under the aerobic condition. The height at each position indicates the number of DNA fragments overlapping that position. The *cydA* promoter contains five known 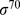 binding sites separated by 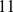 to 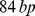 [25]. As evidenced in Figure 1B, coverage plots for PET and SET appear almost indistinguishable visually. To further understand the appearance of peaks that multiple binding events in this region would result in, we simulated PET and SET data with parameters matching to those of this region. Figures S1A, B, C in Text S1 display SET and PET coverage plots of this region when it harbors one and three binding events. These plots support that when binding events are in close proximity with distances less than the average library size, they appear as uni-modal peaks regardless of the library preparation protocol (Figure S1C in Text S1). We next evaluated two peak callers, MACS [9] and MOSAiCS [10], both of which are specifically developed for SET data, on our SET and PET experimental datasets (Table S1 in Text S1). Both methods identified broad regions and the median widths of MACS peaks were 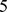 to 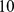 times larger than those of the MOSAiCS peaks. Detailed comparison of the MACS and MOSAiCS peaks revealed that each MACS peak on average has 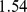 to 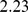 MOSAiCS peaks (Table S2 in Text S1). Next, we evaluated the number of annotated 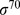 binding events from RegulonDB [25] (http://regulondb.ccg.unam.mx/) in each of the MACS and MOSAiCS peaks and found that MACS peaks, on average, had 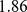 to 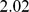 annotated binding events whereas MOSAiCS peaks had 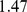 to 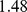. Overall, we did not observe any differences in the peak widths of the PET and SET assays with MOSAiCS whereas MACS peaks from PET data tended to be wider than those of the SET data. These findings indicate that the potential advantages of the PET assay for elucidating closely located binding sites are not simply revealed from visual inspection and by analysis with methods developed specifically for SET data. Hence, deciphering the advantages of PET over SET for high resolution binding site identification warrants a statistical assessment. Next, we developed a generative probabilistic model and an accompanying algorithm, dPeak, that can specifically utilize local read distributions from SET and PET assays. This algorithm enabled unbiased evaluation of the SET and PET assays using our *E. coli* SET and PET ChIP-Seq data.

### Analytical framework of the dPeak algorithm

dPeak requires data in the form of genomic coordinates of paired reads (for PET) or genomic coordinates of reads and their strands (for SET) obtained from mapping to a reference genome. For computational efficiency, dPeak first identifies candidate regions (i.e., peaks) that contain at least one binding event and considers each candidate region separately for the prediction of number and locations of binding events (the first step of Figure 1C). Either two-sample (using both ChIP and control input samples) or one-sample (only using ChIP sample when a control sample is lacking) analysis can be used to identify candidate regions. For this purpose, we utilize the MOSAiCS algorithm [10] which produced narrower peaks than the MACS algorithm [9] in our ChIP-Seq datasets (Table S1 in Text S1).

In each candidate region, we model read positions as originating from a mixture of multiple binding events and a background component (the third step of Figure 1C). dPeak infers the number of binding events and the read sets corresponding to each binding event within each region. It iterates the following two steps for each candidate region. First, it assigns each read to a binding event or background, based on the positions and strengths of the binding events. Then, the position and strength of each binding event are updated using its assigned reads. In practice, the number of binding events in each candidate region is unknown *a priori*. Hence, we consider models with different numbers of binding events and choose the optimal number using Bayesian information criterion (BIC) [26]. We constructed generative probabilistic models for binding event components and a background component for each of the PET and SET data by careful exploratory analyses of multiple experimental ChIP-Seq datasets. Diagnostic plots of the fitted models (Figure S3 in Text S1) indicate that the dPeak model fits ChIP-Seq data well.

dPeak has two unique features compared to other peak deconvolution algorithms (Table S3 in Text S1). First, it accommodates both SET and PET data and explicitly utilizes specific features of both types. Second, it incorporates a background component that accommodates reads due to non-specific binding. Consideration of non-specific binding is critical because the degree of non-specific binding becomes more significant as the sequencing depths get larger. An additional unique feature of dPeak is the treatment of unknown library size for SET data. As discussed earlier, to account for unknown library size, each read is either extended to or shifted by an estimate of the library size in most peak calling algorithms [20]. This estimate is often specified by users [7], [10] or estimated from ChIP-Seq data [9], [11]. Currently available algorithms with the exception of PICS use only one extension/shift estimate for all the regions in the genome. However, our exploratory analysis of real ChIP-Seq data and the empirical distribution of the library size from PET data (Figure S2A in Text S1) indicate that using single extension/shift length might be suboptimal for peak calling (data not shown). In order to address this issue, dPeak estimates optimal extension/shift length for each candidate region. Comparison of empirical distribution of the library size from PET data with the estimates of the region-specific extension/shift lengths indicates that dPeak estimation procedure handles the heterogeneity of the peak-specific library sizes well (Figures S2B, C, D in Text S1). This advancement ensures that dPeak is well tuned for deconvolving SET peaks, which then enables an unbiased computational comparison between the SET and PET assays.

### dPeak outperforms competing methods in discovering closely spaced binding events from SET ChIP-Seq data

We compared dPeak with two competing algorithms, GPS [22] and PICS [11], for analysis of SET ChIP-Seq data. We did not include the CSDeconv algorithm [21] in this comparison because it is computationally several orders of magnitude slower than the algorithms considered here. We utilized the synthetic ChIP-Seq data which was previously used to evaluate deconvolution algorithms [22]. In this synthetic data, binding events were generated by spiking in reads from predicted CTCF binding events at predefined intervals [22] without explicitly implanting binding sequence motifs. Therefore, we also excluded GEM [23], which capitalizes on motif discovery to infer positions of binding events, from this comparison and used additional computational experiments below to perform comparisons with GEM. The synthetic data from [22] consisted of 1,000 joint (i.e., close proximity) binding events, each with two events, and 20,000 single binding events. We assessed performances of algorithms on these two sets separately.

**Figure 2:**
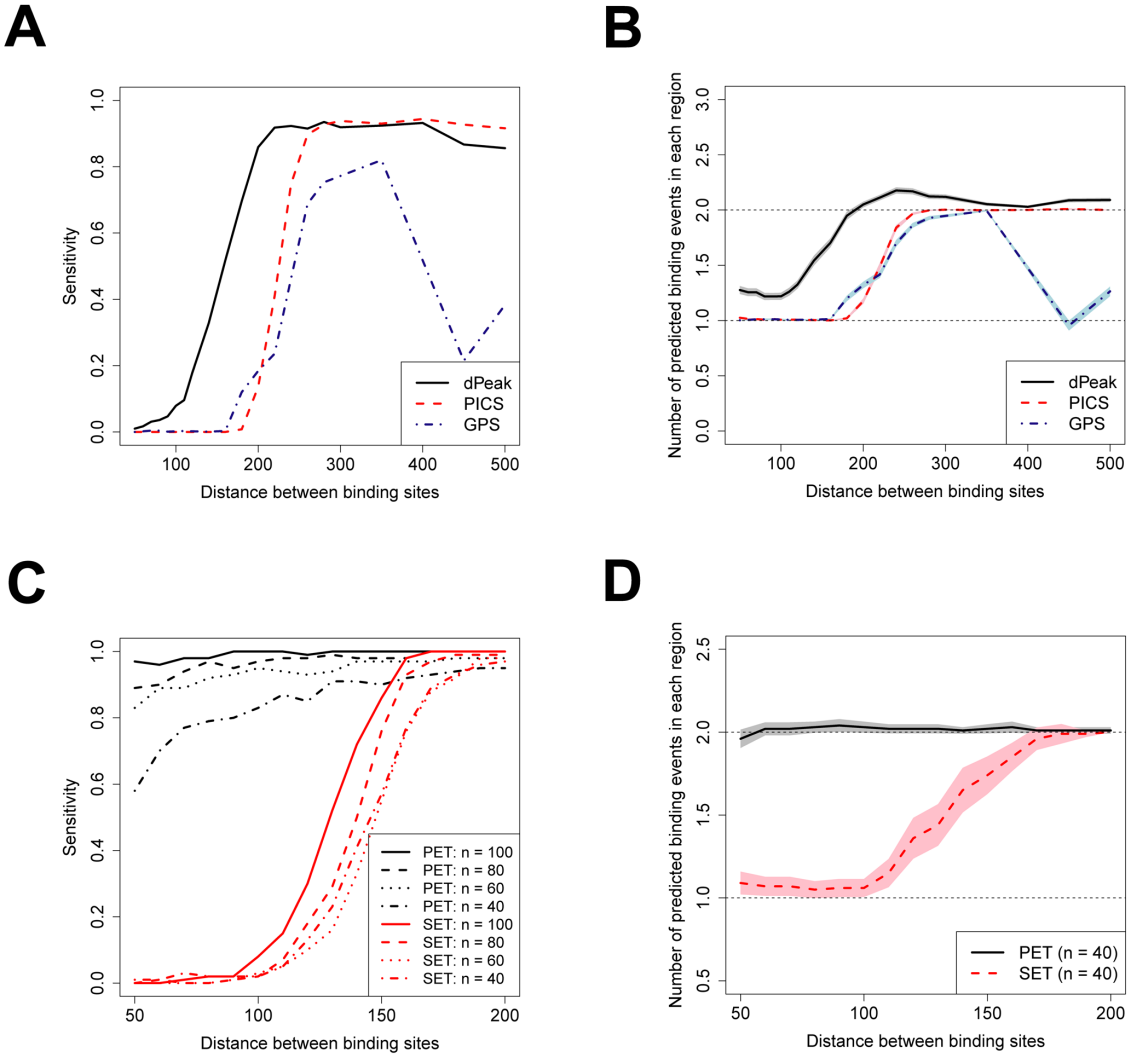
Sensitivity and positive predictive value comparisons of high resolution binding site identification algorithms and dPeak performance on PET vs. SET data. (A, B) Comparison of dPeak with PICS and GPS in computational experiments designed for the GPS algorithm. (A) dPeak has higher sensitivity than both PICS and GPS for SET ChIP-Seq data, especially when the distance between binding events is less than the library size. (B) When there are two true binding events in each region, dPeak on average generates more than one prediction and results in a comparable positive predictive value to those of PICS and GPS. PICS and GPS on average generate only one prediction when the distance between binding events is less than the library size. Shaded areas around each line indicate confidence intervals. (C, D) Comparison of PET and SET assays with dPeak. (C) For SET ChIP-Seq data, the sensitivity of dPeak significantly decreases as the distance between the locations of the events decreases. In contrast, sensitivity from PET ChIP-Seq data is robust to the distance between closely located binding events. The sensitivity for both PET and SET data also decreases as number of reads decreases. (D) dPeak on average predicts two binding events with PET ChIP-Seq data at any distance between the two joint binding events and results in excellent positive predictive value. SET ChIP-Seq data predicts significantly fewer number of binding events as the distance between binding sites decreases. In (C) and (D), n indicates number of reads corresponding to each binding event and 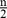 DNA fragments are used for PET data to match the number of reads between PET and SET data. (D) shows the case that 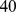 reads correspond to each binding event and results are similar for other number of reads. Shaded areas around each line indicate confidence intervals.

Figure 2A shows the sensitivity of each algorithm at different distances between the joint binding events. Here, sensitivity is the proportion of regions for which both of the two true binding events are correctly identified. dPeak outperforms other methods across all considered distances between the joint binding events and especially for closely located binding events separated by less than the average library size of 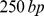. When the distance between the joint binding events is about 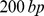, dPeak is able to identify both binding events in 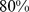 of the regions whereas neither PICS nor GPS can detect both binding events in more than 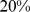. Further investigation indicates that PICS merges closely spaced binding events into one event too often (Figure S4 in Text S1). We also found that GPS estimates the peak shape incorrectly when ChIP-Seq data harbors many closely located binding events (Figure S5 in Text S1). Furthermore, the sensitivity of GPS also decreases significantly when the distance between joint binding events increases. A closer look at the results reveals that GPS filters out too many predictions for joint binding events.

To ensure that increased sensitivity of dPeak is not a result of increased number of false predictions, we evaluated positive predictive value (fraction of predictions that are correct) of each method. Specifically, we plotted the number of binding events predicted by each algorithm at different distances between the joint binding events in Figure 2B. Since there are two true binding events in each region, two predictions at every distance correspond to perfect positive predictive value. dPeak on average generates more than one prediction and does not over-estimate the number of binding events when the distance between joint events is less than the average library size. This result confirms that the higher sensitivity of dPeak in Figure 2A is not due to increased number of predictions. In contrast, PICS and GPS on average generate only one prediction for closely located binding events, which recapitulates the conclusions from Figure 2A. In summary, dPeak outperforms state-of-the-art deconvolution methods across different distances between joint binding events, especially when the distance between the binding events is less than the average library size.

Next, we evaluated the sensitivity and positive predictive value of the three methods on 20,000 candidate regions with a single binding event using the additional synthetic data from [22] (Table S4 in Text S1). Average number of predictions per region with at least one predicted binding event and the corresponding standard errors are as follows: dPeak 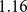 (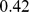), PICS 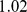 (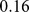), GPS 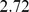 (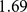). Overall, dPeak slightly over-estimates the number of binding events for regions with a single binding event, and hence PICS is slightly better than dPeak in positive predictive value for these regions. However, as revealed by our joint event analysis, this conservative approach of PICS severely under-estimates the number of binding events when multiple events reside closely. In contrast, GPS significantly under-estimates the number of binding events for the regions with a single binding event since it filters out too many predictions and does not result in a prediction for 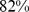 of the regions. In addition, it over-estimates the number of binding events across regions for which it produces at least one prediction. Comparisons in these two scenarios with and without joint binding events indicate that dPeak strikes a good balance between sensitivity and positive predictive value for both cases.

### PET is more powerful than SET for resolving closely spaced binding events

Once we developed dPeak as a high resolution peak detection method for both SET and PET data, we implemented simulation studies to evaluate the PET and SET assays for resolving closely spaced binding events in an unbiased manner. Although SIPeS [24] supports PET ChIP-Seq data, we excluded it from the comparison of PET and SET ChIP-Seq datasets due to its poor performance (Section 16 of Text S1). We generated 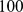 simulated PET and SET ChIP-Seq data with two closely spaced binding events and evaluated the predictions of these two data types with dPeak (Section 11 of Text S1; Figure S7 in Text S1).

Figure 2C plots the sensitivity of dPeak as a function of distance between the joint binding events and number of reads for both the PET and SET settings. Note that we evaluated sensitivity up to the distance of 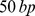 because we used 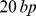 windows to determine whether a binding event is correctly identified and as a result, results for the distance less than 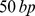 could be misleading. When the distance between the events is at least as large as the average library size (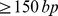), the sensitivity using PET and SET data are comparable. However, as the distance between joint binding events decreases, the sensitivity using SET data decreases significantly. In contrast, PET ChIP-Seq retains its high sensitivity even for binding events that are located as close as 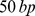. As the number of reads decreases, sensitivity for both PET and SET data decreases. When there are only 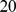 DNA fragments (i.e., 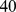 reads) per binding event, sensitivity for PET data also decreases as the distance between joint binding events decreases. However, even in this case, sensitivity of PET data is still significantly higher than that of SET data with much higher number of reads. Figure 2D displays the number of binding events predicted by dPeak at different distances between joint binding events when 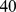 reads correspond to each binding event for both PET and SET data and evaluates positive predictive value. Results are similar for higher number of reads (data not shown). With PET ChIP-Seq, dPeak accurately chooses the number of binding events by BIC out of a maximum of five binding events at any distance between the joint binding events. In contrast, SET ChIP-Seq predicts less than two binding events when the distance between the events is less than 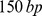.

We present additional simulation results in Section 10 of (Figure S6 in Text S1). These simulations reveal that even for cases with single binding events, PET has a slight advantage over SET because it predicts the location of the binding event more accurately. Specifically, PET data always provides higher resolution compared to SET data regardless of the strength of the binding event, which we measure by the number of DNA fragments associated with the event. For example, for a binding event with 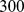 DNA fragments, the average distance between the predicted and true binding events is 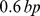 with a standard deviation of 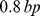 in the PET data whereas it is 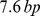 with a standard deviation of 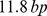 in the SET data. Note that although this simulation procedure is based on the assumptions of dPeak model for PET data, our exploratory analysis and goodness of fit (Figure S3A in Text S1) show that these assumptions hold well in the real PET ChIP-Seq data and therefore, these results have significant practical implications for real ChIP-Seq data.

### Analytical investigation with the dPeak generative model explains the difference in sensitivity between PET and SET data

**Figure 3:**
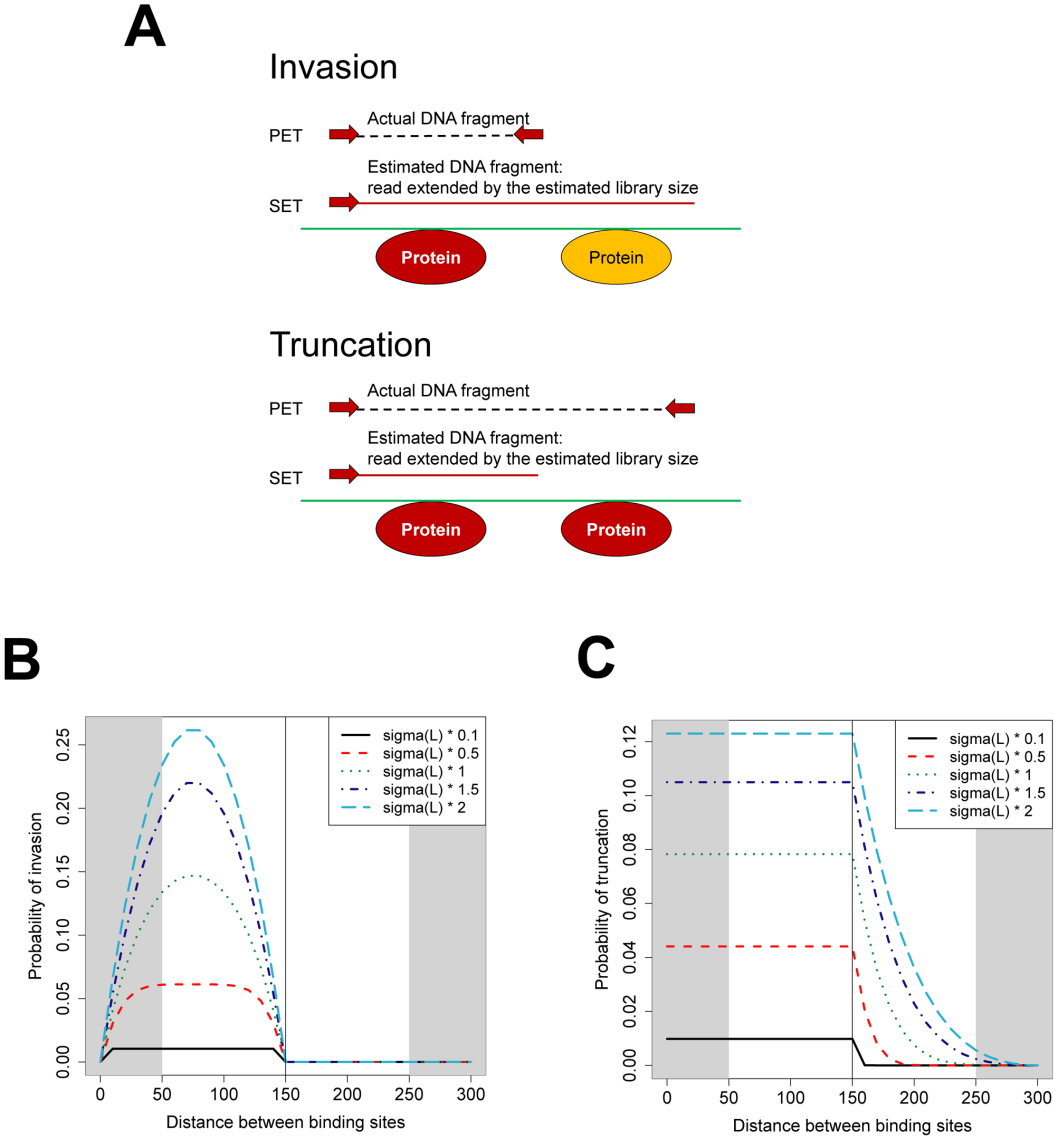
Illustration of loss of information in SET assay compared to PET assay. (A) Concepts of *invasion* (top diagram) and *truncation* (bottom diagram). In each diagram, the first and second lines indicate PET and SET ChIP-Seq data, respectively. Red horizontal line depicts estimated library size in the SET data. Red circles denote the protein binding event that the read corresponds to. In the case of invasion, this read becomes uninformative regarding the protein binding event whereas with truncation, the read provides incorrect information about the protein binding event. (B) Probability of invasion as a function of distance between binding sites based on the dPeak generative model. (C) Probability of truncation as a function of distance between binding sites based on the dPeak generative model. In (B) and (C), *sigma(L)* refers to estimated standard deviation of the library size distribution in 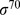 PET ChIP-Seq data and sigma(L) * a indicates that the simulation uses standard deviation of sigma(L) * a to generate library size. Unshaded areas depict typical range of library sizes.

Lower sensitivity of the SET compared to PET data is mainly driven by the loss of information due to unknown library size. We describe this information loss by two concepts named *invasion* and *truncation* (Figure 3A). Top diagram of Figure 3A depicts two closely spaced binding events and a DNA fragment that is informative for the first binding event (in red) in the PET data. *Invasion* refers to over-estimation of the library size and extension of the read to a length longer than the true one. Equivalently, in the shifting procedure, this corresponds to shifting the read more than necessary. As a result, the read extended to the estimated library size covers both of the closely spaced binding events in the SET data and becomes uninformative or less informative for the binding event it corresponds to. Bottom diagram of Figure 3A also depicts two closely spaced binding events and illustrates *truncation* which we define as under-estimation of the library size. In this case, the displayed DNA fragment is long and spans both binding events (in red). Therefore, it contributes to estimation of both binding events in the PET data. In contrast, the read extended to estimated library size only covers the first binding event in the SET data and, as a result, its contribution to the first binding event is overestimated whereas its contribution to the second binding event is underestimated. We evaluated the frequency by which fragments with invasion and truncation arise in SET data with a simulation study. Our results (Table S5 in Text S1) indicate that as high as 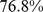 and 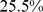 of the fragments for a typical peak region can be subject to invasion and truncation with the SET assay.

Figures 3B, C display the probabilities of invasion and truncation, respectively, of a DNA fragment as a function of the distance between binding events and the variance of the library size. The analytical calculations are based on the dPeak generative model (Section 12 of Text S1). Probabilities of invasion and truncation are higher for closely spaced binding events, especially when the library size is shorter than the estimated library size (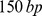 in this case). In Figure 3B, the probability of invasion decreases for very closely spaced binding events, i.e., when the distance between two binding events is less than 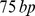. As the distance between the binding events decreases, most DNA fragments cover both binding events and the configuration in the first diagram of Figure 3A is unlikely to occur. Hence, there is already insufficient information to predict two binding events even in PET data and relative loss of information (i.e., invasion) in SET data is insignificant. These concepts describe how information on binding events can be lost or distorted by the incorrect estimation of the library size in the SET data. Analytical calculations based on the dPeak generative model show that invasion and truncation influence closely located binding events the most, especially when the library size is not tightly controlled, i.e., exhibit large variation (Figures 3B, C).

### dPeak analysis of 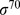 PET ChIP-Seq data identifies significantly more RegulonDB supported 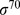 binding events than the analysis of SET ChIP-Seq data

**Figure 4:**
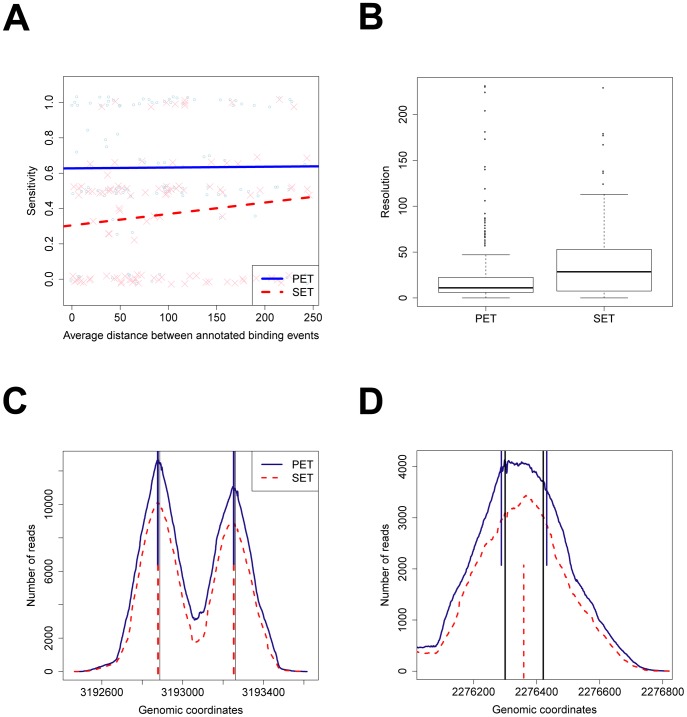
dPeak analyses and evaluations of 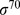 PET and SET ChIP-Seq data based on RegulonDB annotated 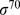 factor binding sites. (A) The numbers of correctly identified binding sites are plotted as a function of the distances between the RegulonDB reported binding events. Linear lines (solid for PET, dashed for SET) through the data points depict general trends. (B) Resolution comparisons of the predictions for the regions with a single annotated binding event. (C, D) PET (blue) and SET (red) coverage plots for representative examples of predicted 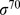 binding sites. Blue and red dotted vertical lines indicate predictions using PET and SET data, respectively. Black solid vertical lines indicate the annotated binding sites in (C) and experimentally validated binding sites in (D).

We compared the performance of PET and SET sequencing for 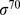 factor under the aerobic condition by generating a ‘quasi-SET data’ by randomly sampling one of the two ends of each paired reads in PET data and comparing binding events identified from both sets. In order to match number of reads with SET data for fair comparison, only the half number of paired reads was used to construct PET data. Comparison with the quasi-SET data controlled for the differences in the sequencing depths of the original PET and SET samples in addition to the biological variation of the replicates. We then evaluated the dPeak predictions from the PET and SET analyses using the 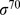 factor binding site annotations in the RegulonDB database as a gold standard. Because a significant number of promoter regions lack RegulonDB annotations, we evaluated the sensitivity based on the regions that contain at least one annotated binding site. This corresponds to 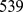 binding sites in 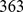 candidate regions that MOSAiCS identified. Of these 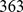 regions, 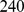 harbor only a single annotated binding event. For the regions with more than one annotated binding event, the average distance between binding events is 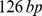. dPeak analysis of the SET data identifies only 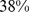 of the 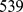 annotated binding events. In contrast, analysis of PET data with dPeak detects 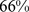 of the annotated binding sites. Figure 4A displays average sensitivity as a function of the average distance between annotated binding events for the regions with at least two RegulonDB annotations. A linear line is superimposed to capture the trend for both data types. Notably, the lower sensitivity of SET compared to PET is mainly due to closely located binding events.

We also compared prediction accuracies of the PET and SET assays for the 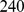 regions that harbor a single annotated binding event. Figure 4B displays resolutions, which we define as the minimum of distances between predicted and annotated positions of binding events, achieved by the PET and SET assays. Median resolutions are 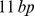 (IQR = 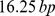) and 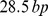 (IQR = 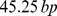) for PET and SET, respectively. This result indicates that positions of binding events can be more accurately predicted with the PET assay compared to SET even for regions with a single binding event.

**Figure 5:**
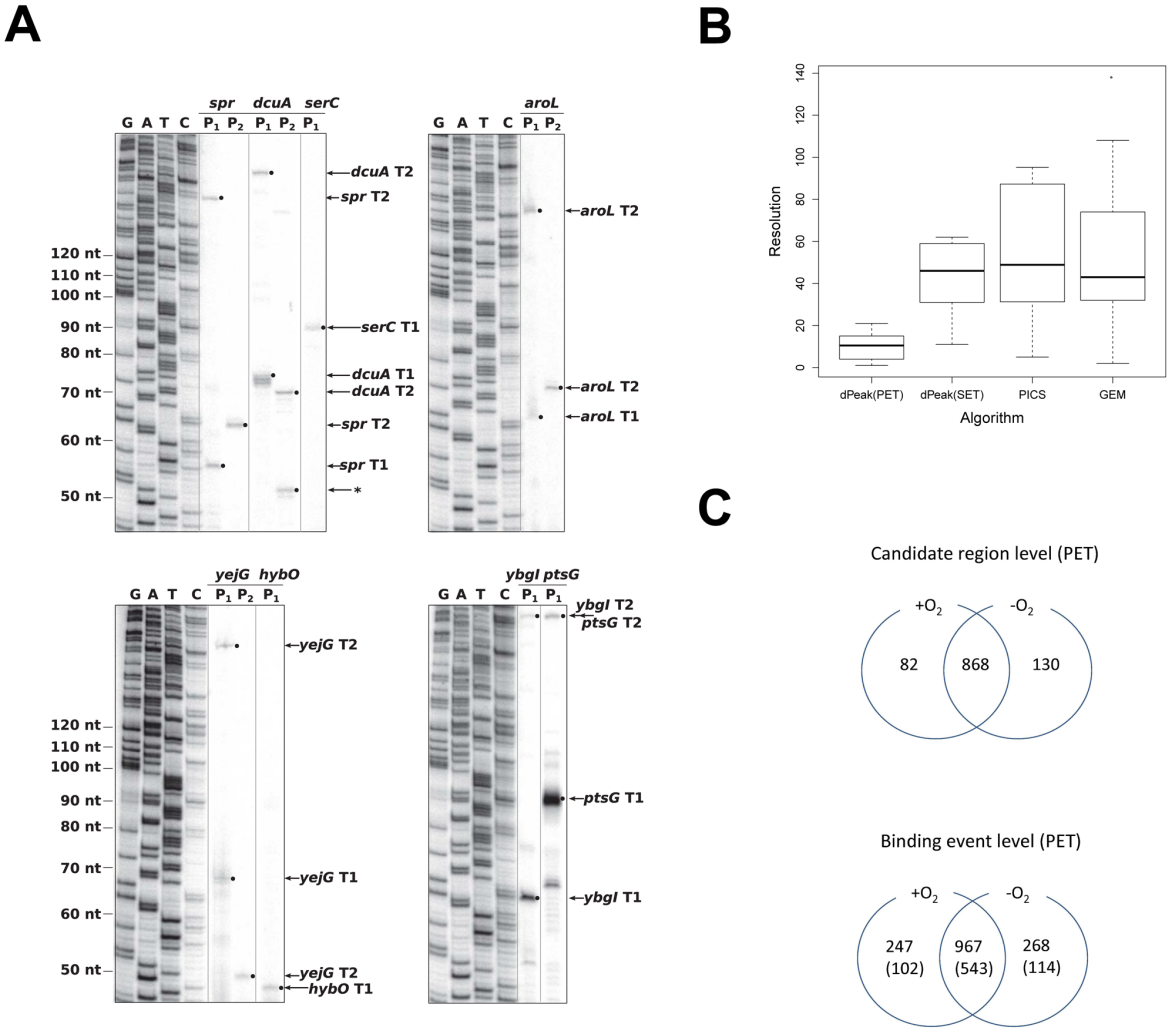
Experimental validation and analysis of differential occupancy using dPeak. (A) Validation of a subset of transcription start site predictions using primer extension. Primers (Table S7 in Text S1) complementary to the mRNA sequence 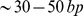 downstream of each predicted start site were 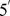 end labeled 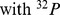 and 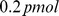 was used for each 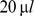 assay. RNA was isolated from either aerobic (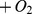) or anaerobic (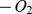) conditions. The sequencing ladders (G, A, T and C) were generated by dideoxy sequencing. Small arrows and filled circles depict the primer extension products. In addition to *dcuA* 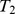, a second, less abundant primer extension product (*) was identified with *dcuA* 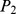. Since this product was not identified with *dcuA* 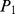, it is possible that it corresponds to the start site of an sRNA which terminates upstream of the priming location of 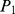. (B) Resolution comparison of the high resolution binding site identification algorithms, using experimentally validated sites as a gold standard (extended version in Figure S9C in Text S1). (C) Summary of the analyses of 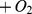 and 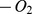 PET ChIP-Seq data. The 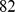, 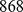, and 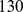 candidate regions (the first diagram) cover 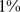, 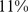, and 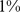 of the *E. coli* genome, respectively. In the bottom diagram, the numbers in parentheses depict the set of binding events that were independently validated with predictions from the analysis of biological replicate SET ChIP-Seq.

To further examine the accuracy of the 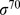 dPeak predictions, primer extension analysis was performed to map the transcription start site for eight genes (Figures S10–S13 in Text S1; Table S7 in Text S1). dPeak analysis of the PET ChIP-Seq data predicts two closely spaced 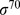 binding sites in the upstream of each of these eight genes with the distance between predictions ranging 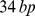 to 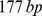. Seven of these predictions are not annotated in RegulonDB and thus represent potential novel transcription start sites. A transcription start site was detected within 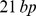 of 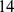 (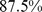) of these 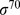 binding site predictions (Figure 5A and Table 1), further supporting the accuracy of the dPeak PET predictions.

**Table 1:** Experimental validation of the binding events predicted by dPeak analysis of 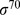 PET ChIP-Seq data.

Gene^a^	Predicted position	True position^b^	Distance	Primer^b^	Condition^c^
*yejG*	2,276,288	2,276,299	11	*P*_1_	Aerobic
*yejG*	2,276,432	2,276,419	13	*P*_2_	Aerobic
*spr*	2,267,945	2,267,942	3	*P*_1_	Aerobic
*spr*	2,267,825	2,267,833	8	*P*_2_	Aerobic
*dcuA*	4,364,876	4,364,866	10	*P*_1_	Anaerobic
*dcuA*	4,364,975	4,364,974	1	*P*_2_	Anaerobic
*aroL*	405,583	405,579	4	*P*_1_	Anaerobic
*aroL*	405,489	405,504	15	*P*_2_	Anaerobic
*serC*	956,823	956,802	21	*P*_1_	Aerobic
*serC*	956,789	(Not validated)	N/A		Aerobic
*hybO*	3,144,382	3,144,385	3	*P*_1_	Anaerobic
*hybO*	3,144,438	(Not validated)	N/A		Anaerobic
*ybgI*	742,036	742,030	6	*P*_1_	Aerobic
*ybgI*	741,859	741,874^d^	15	*P*_1_	Aerobic
*ptsG*	1,157,005	1,156,989	16	*P*_1_	Aerobic
*ptsG*	1,156,866	1,156,849^d^	17	*P*_1_	Aerobic

(a) The genes with promoters harboring the predicted binding events.

(b) The true positions were determined by primer extension experiments (Figure 5A).

(c) The conditions under which binding events are validated.

(d) We report results based on the RegulonDB annotations for *ybgI* and *ptsG* genes as the primer extension products for these genes were too large to accurately map with the sequencing ladder.

We treated these 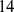 validated sites as a gold standard and evaluated the performance of each deconvolution algorithm for these regions. Figure 5B depicts that dPeak with PET ChIP-Seq data attains significantly higher resolution compared to SET-based analysis regardless of the deconvolution algorithm used (*p*-values of paired *t*-tests between dPeak using PET data and each of the other methods using SET data are 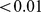). dPeak with SET ChIP-Seq data has a resolution comparable to or better than those of the competing algorithms. GPS is not included in this plot because it provides significantly worse resolution compared to other methods (Figure S9C in Text S1). Genome-wide comparisons using the RegulonDB transcription start site annotations as a gold standard also lead to a similar conclusion, supporting the notion that PET-analysis with dPeak provides the best resolution (Figures S9A, B in Text S1).

Figures 4C and 4D display two representative peak regions from these analyses. Figure 4C illustrates two binding events in the promoter regions of *sibD* and *sibE* genes separated by 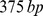. In this case, two peaks are easily distinguishable just by visual inspection and the predictions using both PET and SET data are comparably accurate. Note that although these two binding events are visually distinguishable, standard applications of MACS and MOSAiCS identify this region as a single peak. Widths of MOSAiCS and MACS peaks for this region are 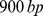 and 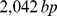, respectively. MACS identifies the position of the right binding event as the “summit” of this region (position 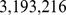). Figure 4D displays the promoter region of *yejG* gene, where the distance between the two experimentally validated binding events is only 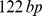. In this case, dPeak application to PET data correctly predicts the number of binding events as two and identifies the locations of these events within 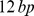 of the validated sites. In contrast, all of the SET-based analyses with the deconvolution algorithms (PICS, GPS, GEM) incorrectly predict one binding event located in the middle of the two experimentally validated binding sites.

### dPeak analysis of *E. coli* 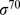 PET ChIP-Seq data identifies closely located binding sites that are differentially occupied between aerobic and anaerobic conditions

High resolution identification of binding sites is especially important for differential occupancy analysis where a protein of interest is profiled under different conditions. Given the high agreement between the dPeak algorithm and experimentally validated transcription start sites at a subset of promoter regions, we set out to identify differential promoter usage between the aerobic and anaerobic growth conditions by profiling the *E. coli* 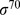 factor. Results from the dPeak analysis of the aerobic and anaerobic PET data are summarized in Figure 5C both in the region (i.e., peak) and binding event levels. We identified 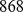 peaks and 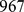 dPeak binding events that were common between the 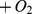 and 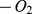 conditions. Interestingly, only 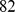 peaks were unique to the 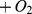 condition but dPeak analysis identified 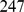 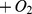-specific binding events. Similarly, we identified 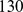 peaks unique to the 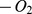 condition while dPeak analysis resulted in 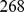 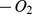-specific binding events. We used the SET ChIP-Seq data from additional biological replicates under both conditions as independent validation of the results. This independent validation using SET data identified 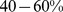 of the binding events identified by dPeak using PET ChIP-Seq data (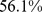 of the common events, 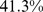 of the 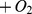-specific binding events and 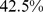 of the 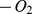-specific binding events). Table S8 in Text S1 further summarizes these results by cross-tabulating the number of predicted binding events in each peak across the two conditions. It illustrates that there are indeed many peaks with at least one binding event in each condition and different number of binding events across the two conditions. Figure S14 in displays an example of closely located binding sites that are differentially occupied between aerobic and anaerobic conditions in 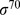 PET ChIP-Seq data. These results suggest that dPeak analysis identified many unique 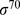 binding events that could not be differentiated in the peak-level analysis.

## Discussion

High resolution identification of binding sites with ChIP-Seq has profound effects for studying protein-DNA interactions in prokaryotic genomes and differential occupancy. We evaluated PET and SET ChIP-Seq assays and illustrated that PET has considerably more power for deciphering locations of closely spaced binding events. Our data-driven computational experiments indicate that when the distance between binding events gets smaller than the average library size, SET analysis have notably less power than the PET analysis. Furthermore, PET provides better resolution than SET even when a region harbors a single binding event. We developed and evaluated the dPeak algorithm, a model-based approach to identify protein binding sites in high resolution, with data-driven computational experiments and experimental validation. dPeak is currently the only algorithm that can utilize both PET and SET ChIP-Seq data and can accommodate high levels of non-specific binding apparent in deeply sequenced ChIP samples (Table S3 in Text S1). Our data-driven computational experiments and computational analysis of experimentally validated 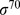 binding sites indicate that it significantly outperforms the currently available PET ChIP-Seq peak finder SIPeS [24]. Application of dPeak to *E. coli* 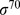 ChIP-Seq data under aerobic and anaerobic conditions revealed that although many peaks identified by standard application of popular peak finders might appear as common between the two conditions, a considerable percentage of these may harbor condition-specific binding events. The high-resolution 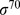 binding sites identified by dPeak could be combined with start-site mapping or consensus-sequence identification to assign transcriptional orientation to the 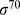 binding sites.

The advantages of using the dPeak algorithm are not limited to the study of prokaryotic genomes. Applications in eukaryotic genomes include identification of the exact locations of binding motifs when multiple closely located consensus sequences reside in a peak region, studies of *cis* regulatory modules (CRM), and refining consensus sequences. Figure S16 in Text S1 displays an example application of dPeak for differentiating among multiple closely located GATA1 binding sites with consensus WGATAR within a ChIP-Seq peak region critical for erythroid differentiation in mouse embryonic stem cells (data from [27]). CRM studies investigate relationships between spatial configurations of binding sites of multiple transcription factors and gene expression. Relative orders, positions, and distances of binding sites of multiple factors and their relative strengths are key factors in CRM studies [28]. Because dPeak facilitates identification of binding sites of transcription factors in high resolution from ChIP-Seq data, it can enable construction of complex interaction networks among diverse factors across multiple growth conditions.

We evaluated the performance of dPeak on eukaryotic genome ChIP-Seq data that GPS and PICS were optimized for. Figure S17 in Text S1 shows the performance comparison results for transcription factor GABPA profiled in GM12878 cell line from the ENCODE database. It indicates that dPeak performs comparable to or outperforms GPS and PICS. In the case of sequence-specific factors with well-conserved motifs such as the GABPA factor, we observed that dPeak prediction can be further improved in a straightforward way by incorporating sequence information. Figure S17 in Text S1 illustrates that dPeak with incorporated sequence information performs comparable to GEM and identifies the GABPA binding sites with high accuracy.

Recently, ChIP-exo assay [29], a modified ChIP-Seq protocol using exonuclease, has been proposed as a way of experimentally attaining higher resolution in protein binding site identification. Because the ChIP-exo protocol is new and relatively laborious, there are not yet many publicly available ChIP-exo datasets. We utilized ChIP-exo of CTCF factor in human HeLa-S3 cell line [29] and compared their binding event predictions with dPeak predictions on SET ChIP-Seq data of CTCF in the same cell line. Figure S18 in Text S1 illustrates that dPeak using SET ChIP-Seq data provides higher resolution than ChIP-exo data and that dPeak can be readily utilized for ChIP-exo data analysis. Furthermore, it also indicates that dPeak performs comparable to or outperforms currently available methods such as GPS and GEM for both ChIP-exo and SET ChIP-Seq data. Although the real power of the ChIP-exo technique will be revealed as more ChIP-exo datasets are produced and compared with ChIP-Seq datasets, our results with the currently available data suggest that analyzing ChIP-Seq data with powerful deconvolution methods such as dPeak might perform as well as ChIP-exo.

We implemented dPeak as an R package named dPeak. dPeak utilizes the fast estimation algorithm we developed and parallel computing. Analysis of the 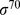 data (∼1,000 candidate regions, each with ∼2,300 reads on average) using our current sub-optimal implementation of dPeak takes about 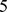 minutes using 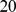 CPUs (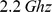) when up to 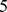 binding events are allowed in each candidate region, while it takes about 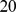 minutes to run PICS and GPS (also using 20 CPUs). Similarly, analysis of human ENCODE POL2-H1ESC data (∼14,000 candidate regions, each with 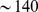 reads on average) takes about 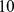 minutes for dPeak, while it takes 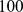 and 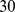 minutes for GPS and PICS, respectively. dPeak is currently available at http://www.stat.wisc.edu/ ~chungdon/dpeak/ and will be contributed to public repositories such as Bioconductor [30] and Galaxy Tool Shed [31] upon publication.

## Materials and Methods

### Growth conditions

All strains were grown in MOPS minimal medium supplemented with 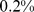 glucose [32] at 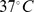 and sparged with a gas mix of 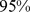 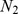 and 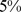 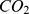 (anaerobic) or 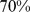 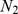, 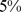 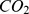, and 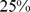 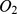 (aerobic). Cells were harvested during mid-log growth (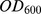 of 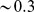 using a Perkin Elmer Lambda 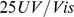 Spectrophotometer). WT *E. coli* K-12 MG1655 (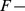, 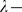, 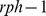) was used for the experiments (Kiley lab stock).

### ChIP experiments

ChIP assays were performed as previously described [33], except that the glycine, the formaldehyde, and the sodium phosphate mix were sparged with argon gas for 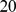 minutes before use to maintain anaerobic conditions when required. Samples were immunoprecipitated using 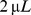 of RNA Polymerase 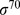 antibody from NeoClone (W0004).

### Library preparation, sequencing, and mapping of sequencing reads

For ChIP-Seq experiments, 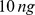 of immunoprecipitated and purified DNA fragments from the aerobic and anaerobic 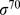 samples (one biological sample for both aerobic and anaerobic growth conditions), along with 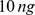 of input control (two biological replicates for anaerobic Input and one biological sample for aerobic Input), were submitted to the University of Wisconsin-Madison DNA Sequencing Facility for ChIP-Seq library preparation. Samples were sheared to 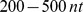 during the IP process to facilitate library preparation. All libraries were generated using reagents from the Illumina Paired End Sample Preparation Kit (Illumina) and the Illumina protocol “Preparing Samples for ChIP Sequencing of DNA” (Illumina part # 11257047 RevA) as per the manufacturer's instructions, except products of the ligation reaction were purified by gel electrophoresis using 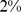 SizeSelect agarose gels (Invitrogen) targeting 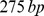 fragments. After library construction and amplification, quality and quantity were assessed using an Agilent DNA 1000 series chip assay (Agilent) and QuantIT PicoGreen dsDNA Kit (Invitrogen), respectively, and libraries were standardized to 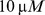. For PET ChIP-Seq data, cluster generation was performed using an Illumina cBot Paired End Cluster Generation Kit (v3). Paired reads, 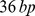 run was performed for each end, using 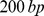 v3 SBS reagents and CASAVA (the Illumina pipeline) v 1.8.2, on the HiSeq2000. For SET ChIP-Seq data, cluster generation was performed using an Illumina cBot Single Read Cluster Generation Kit (v4) and placed on the Illumina cBot. A single read, 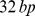 run was performed, using standard 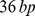 SBS kits (v4) and SCS 2.6 on an Illumina Genome Analyzer IIx. Base calling was performed using the standard Illumina Pipeline version 1.6. Sequence reads were aligned to the published *E. coli* K-12 MG1655 genome (U00096.2) using the software packages SOAP [34] and ELAND (within the Illumina Genome Analyzer Pipeline Software), allowing at most two mismatches. PET experiments yielded 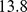 million (M) and 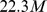 mappable paired 36mer reads and SET yielded 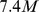 and 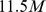 mappable 32mer reads for aerobic and anaerobic conditions, respectively. Control input experiments, generated with SET sequencing, resulted in 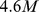 and 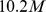 mappable 32mer reads for the aerobic and anaerobic conditions, respectively. Raw and aligned data files are available at ftp://ftp.cs.wisc.edu/pub/users/keles/dPeak and are being processed by GEO for accession number assignment.

### dPeak model

For PET data, if a DNA fragment (paired reads) belongs to 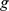-th binding event, we model its leftmost position conditional on its length 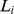 as Uniform distribution between 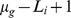 and 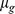, where 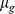 is the position of 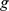-th binding event. Lengths of DNA fragments, 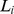, are modeled using the empirical distribution obtained from actual PET data. For SET data, if a read belongs to 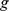-th binding event, we model its 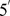 end position conditional on its strand as Normal distribution. Specifically, if a read is in the forward strand, its 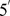 end position is modeled as Normal distribution with mean 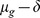 and variance 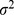. 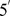 end positions for reverse strand reads are modeled similarly with Normal distribution with mean 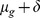 and variance 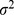. Parameters 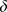 and 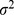 are common to all binding event components in each candidate region. Strands of reads are modeled as Bernoulli distribution. Background reads are assumed to be uniformly distributed over the candidate region that they belong to. Parameters are estimated via the Expectation-Maximization (EM) algorithm [35]. Additional details on the dPeak model and the estimation algorithm for the PET and SET settings are available in Sections 2 and 3 of Text S1.

### Method comparison for SET ChIP-Seq data

We compared the sensitivity and the number of predictions of dPeak with those of PICS [11], GPS [22], and GEM [23]. Sensitivity is the proportion of regions for which both of the two true binding events are correctly identified. A binding event is considered as ‘identified’ if the distance between the actual binding event and the predicted position is less than 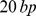. Note that we chose a more stringent criteria than the 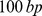 used by GPS for defining true positives because 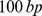 is not high enough resolution for prokaryotic genomes. For the PICS algorithm, we used the R package PICS version 1.10, which is available from Bioconductor (http://www.bioconductor.org/packages/2.10/bioc/html/PICS.html). For the GPS algorithm, we used its Java implementation version 1.1 from http://cgs.csail.mit.edu/gps/. In the performance comparisons using 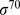 ChIP-Seq data, we also incorporated GEM, a recently modified and extended version of GPS, which incorporates genome sequence of the peaks to improve binding event identification. For the GEM algorithm, we used its Java implementation version 0.9 from http://cgs.csail.mit.edu/gem/. We downloaded the synthetic data used for the method comparisons from http://cgs.csail.mit.edu/gps/ and its description is provided in Supplementary information of the GPS paper [22]. This synthetic data consists of “chrA” with 1,000 regions that harbor two closely spaced binding events and “chrB” to “chrK” with a total of 20,000 regions with a single binding event. We evaluated performances of the methods on joint and single binding event regions separately so that we could assess sensitivity and specificity for each of these cases. Candidate regions for dPeak were identified using the conditional binomial test [6] with a false discovery rate of 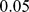 by applying the Benjamini-Hochberg correction [36]. These regions were also explicitly provided to the GPS and GEM algorithms as candidate regions. Candidate regions for PICS were identified using the function segmentReads() in the PICS R package (default parameters). Default tuning parameters were used during model fitting for all the methods.

### Simulation studies to compare PET and SET ChIP-Seq data

We considered distances between binding sites ranging from 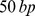 to 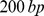 which characterize the typical binding event spacing in *E. coli*. We generated and assigned 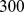 DNA fragments to each of two binding events as follows. For each DNA fragment, we drew the length (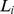) from the distribution of library size, 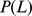, estimated empirically from the actual 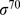 PET ChIP-Seq data and group index (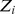) from multinomial distribution with parameters (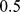, 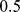). Then, for given a library size and group index (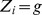), leftmost position of the paired reads (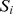) was generated from Uniform distribution between 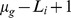 and 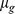, where 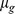 is the position of 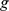-th binding event. Rightmost position was assigned as 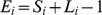. SET data was generated by randomly sampling one of two ends from each of these paired reads. For the SET analysis, average library size was assumed to be 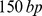. Then, only half of the total number of paired reads was used to construct PET data, in order to match number of reads with SET data for fair comparison. In addition, we randomly assigned 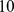 DNA fragments to arbitrary positions within the candidate region to generate non-specific binding (background) reads. The sensitivity and the number of predictions were summarized over 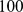 simulated datasets generated by this procedure. A binding event was considered as ‘identified’ if the distance between the binding event and the predicted position is less than 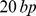. We repeated these PET versus SET analyses by comparing all the PET data with SET data constructed from selecting one of two ends of each read pair and obtained little or no change in the results (data not shown).

### dPeak analysis of 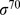 PET and SET ChIP-Seq data

We identified candidate regions, i.e., peaks with at least one binding event, using the MOSAiCS algorithm [10] (two-sample analysis with a false discovery rate of 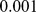). In each candidate region, we fitted the dPeak model, which is a mixture of 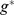 binding event components and one background component (Figure 1C). In the current analysis, up to five binding event components (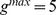) were considered. The optimal number of binding events was chosen with BIC for each candidate region. We utilized top 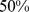 of the predicted binding events from each condition for the comparison between the aerobic and anaerobic conditions. Overall conclusions remained the same when the full set of predicted binding events are considered.

### Primer extension experiments

Total RNA was isolated as previously described [37]. Oligonucleotide primers (Table S7 in Text S1) were labeled at the 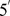 end using [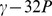]ATP (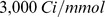) and T4 polynucleotide kinase (Promega) followed by purification with a G25 Sephadex Quick Spin Column (GE). Labeled primer (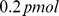) was annealed with 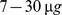 total RNA in 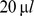 and extended with avian myeloblastosis virus reverse transcriptase (Promega) as described by the manufacturer, except that actinomycin D was present at 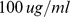 [38]. Primer extension experiments were implemented for *spr* (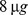 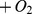 RNA), *dcuA* (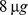 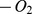 RNA), *serC* (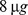 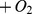 RNA), *aroL* (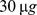 and 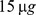 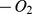 RNA for 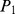 and 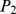, respectively), *yejG* (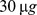 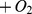 RNA), *hybO* (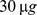 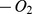 RNA), *ybgI* (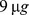 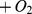 RNA), and *ptsG* (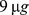 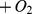 RNA). A dideoxy sequencing ladder was electrophoresed in parallel with the primer extension products on a 8% (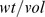) polyacrylamide gel containing 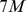 urea. In cases where the transcription start site could be assigned to one of two nucleotides, preference was given to the purine nucleotide.

### Software availability

The dPeak algorithm is implemented as an R package named dpeak and is freely available from http://www.stat.wisc.edu/~chungdon/dpeak/. We will commit dpeak to Bioconductor (http://www.bioconductor.org) and Galaxy Tool Shed (http://toolshed.g2.bx.psu.edu) upon publication.

## Supporting Information

Text S1 **Supplementary methods for “dPeak: High Resolution Identification of Transcription Factor Binding Sites from PET and SET ChIP-Seq Data”.**
(PDF)
