# Supplementary material for: dPeak: High Resolution Identification of Transcription Factor Binding Sites from PET and SET ChIP-Seq Data
Source: PLoS Comput Biol. 2013 Oct 17;9(10):e1003246. doi: 10.1371/journal.pcbi.1003246 (PMC3798280; doi:10.1371/journal.pcbi.1003246)
Supplement: Text S1 — Supplementary methods for “dPeak: High Resolution Identification of Transcription Factor Binding Sites from PET and SET ChIP-Seq Data”. (PDF) [file pcbi.1003246.s001.pdf]

Text S1: Supplementary Methods for "dPeak: High Resolution Identification of Transcription Factor Binding Sites from PET and SET ChIP-Seq Data"

Dongjun Chung[1,#], Dan Park[2], Kevin Myers[2], Jeffrey Grass[3,4], Patricia Kiley[2,4], Robert Landick[3,4,5] & Sündüz Keleş[1,6]

1 Department of Statistics, University of Wisconsin, Madison, WI, U.S.A.
2 Department of Biomolecular Chemistry, University of Wisconsin, Madison, WI, U.S.A.
3 Department of Biochemistry, University of Wisconsin, Madison, WI, U.S.A.
4 Great Lakes Bioenergy Research Center, University of Wisconsin, Madison, WI, U.S.A.
5 Department of Bacteriology, University of Wisconsin, Madison, WI, U.S.A.
6 Department of Biostatistics and Medical Informatics, University of Wisconsin, Madison, WI, U.S.A.
# Current address: Department of Biostatistics, Yale University, New Haven, CT, U.S.A.

1 Analysis of the *E. coli* σ^{70} PET and SET ChIP-Seq Data from Aerobic and Anaerobic Conditions by MACS and MOSAiCS

Using MACS (version 1.3.4) and MOSAiCS (version 1.4.0), we performed two sample analysis of the *E.coli* σ^{70} PET and SET ChIP-Seq data (Table S1). For PET ChIP-Seq data, MACS first finds the best pairs of $5'$ and $3'$ reads from multiple alignment results. Then, only the $5'$ read position is kept for every pair and shifted to its $3'$ direction by $100bp$ without estimation of the shift parameter. Then, the

standard MACS analysis [1] is applied to the processed data. In MOSAiCS, when bin-level data are constructed, each read pair is connected and this connected read pair contributes to all the bins it overlaps. The standard MOSAiCS analysis [2] is applied to this bin-level data. Detailed comparison of the MACS and MOSAiCS peaks reveals that each MACS peak on average has 1.54 to 2.23 MOSAiCS peaks (Table S2).

Experiment	PET		SET	
	MACS	MOSAiCS	MACS	MOSAiCS
$+O_2$	270/3202/22	950/450/11.3	534/2550/34	1023/450/11.3
$-O_2$	132/4327/14	993/450/11.8	469/2890/34	1014/450/11.4

Table S1: Analysis of the PET and SET data with MACS and MOSAiCS. Reported numbers a/b/c refer to a: number of peaks; b: median peak width; c: percent genome coverage.

Experiment	Mean (SD)
PET, $+O_2$	1.82 (0.93)
PET, $-O_2$	2.23 (1.10)
SET, $+O_2$	1.54 (0.80)
SET, $-O_2$	1.65 (0.93)

Table S2: Mean number of MOSAiCS peaks overlapping each MACS peak.

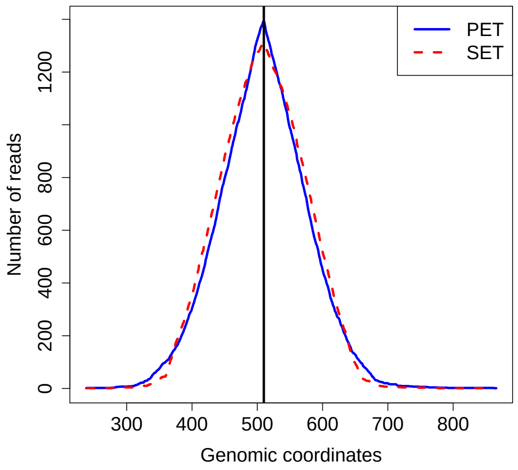

(A)

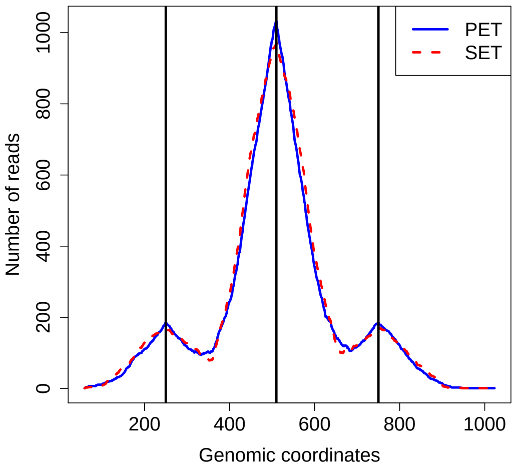

(B)

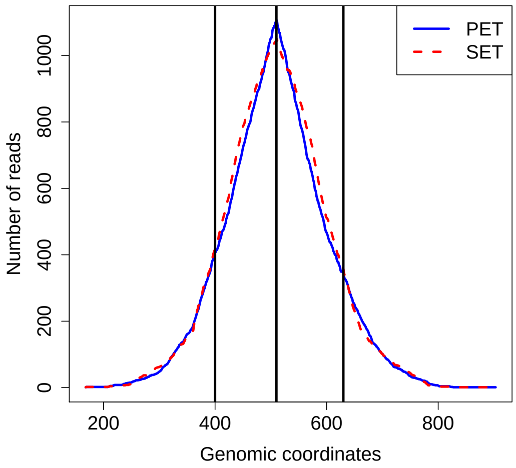

(C)

Figure S1: Coverage plots of simulated read data generated based on *cydA* promoter parameters estimated by dPeak: (A) single binding event; (B, C) three binding events. dPeak analysis of PET data under aerobic conditions generated three binding event predictions for the *cydA* promoter region. Consecutive distances between these binding events are 110*bp* and 120*bp*, respectively. The numbers of DNA fragments corresponding to each event are 180, 1035, and 180 (total of 1395), respectively. (A) One simulated binding event (depicted with the black vertical line) with 1395 reads. (B) Three simulated binding events at locations 250, 510, and 750, and with numbers of reads 180, 1035, and 180. (C) Three simulated binding events at locations 400, 510, and 630, and with numbers of reads 180, 1035, and 180.

2 The dPeak Model

Consider a peak region with n reads (DNA fragments) and let 1 and m denote the start and end positions of the peak region, respectively. Let g^* denote the number of binding events within the region and μ_g be the position of g-th binding event, $g = 1, 2, \cdots, g^*$. Without loss of generality, assume that $1 \leq \mu_1 < \mu_2 < \cdots < \mu_{g^*} \leq m$ for identifiability. In both PET and SET data, a fraction of reads will denote background noise. We assume that background reads are uniformly distributed over the whole candidate region and denote the background component as $g = 0$.

Let π_g denote the strength of g-th binding event, $g = 1, 2, \cdots, g^*$. π_0 indicates degree of non-specific binding in the candidate region. Let Z_i be the group index of i-th DNA fragment and $Z_i \in \{0, 1, 2, \cdots, g^*\}$. For notational convenience, we denote $Z_{ig} = 1\{Z_i = g\}$, where $1\{A\}$ is an indicator function of event A. We assume that $P(Z_i = g) = P(Z_{ig} = 1) = \pi_g$, $g = 0, 1, 2, \cdots, g^*$ and $\sum_{g=0}^{g^*} \pi_g = 1$. Note that the dPeak model allows each DNA fragment to overlap with multiple binding events. The unobserved Z_i variable ensures that each fragment that is not part of the background overlaps with at least one binding event.

2.1 Generative model for paired-end tag (PET) data

Let S_i and L_i be the start position and length of i-th DNA fragment, respectively. If we denote end position of i-th fragment as E_i, then $E_i = S_i + L_i - 1$ by definition. In the PET data, we directly observe S_i and E_i (equivalently, S_i and L_i) for each DNA fragment. Moreover, distribution of library size, $P(L)$, can be empirically estimated from the PET data and hence, we treat $P(L)$ as known. We denote the whole candidate region as $C = \{1 - L_i + 1 \leq S_i \leq m\}$ and the region corresponding to g-th binding event as $B_g = \{\mu_g - L_i + 1 \leq S_i \leq \mu_g\}$. If i-th fragment is generated from g-th binding event ($Z_i = g$), then for given L_i, we assume that S_i is generated from the following Uniform-like distribution:

$$P(s|l; \mu_g, \gamma) = \left[\frac{(1-\gamma)}{l}\right]^{1\{s \in B_g\}} \left[\frac{\gamma}{m-1}\right]^{1\{s \in C \setminus B_g\}},$$

where γ denotes the weight assigned to the area outside of the region corresponding to g-th binding event.

The main purpose to using $P(s|l; \mu_g, \gamma)$ is to make it easier to escape from local maxima during the early iterations of EM algorithm, by making boundaries of B_g "softer" than Uniform distribution. As shown in Section 3.1, γ estimate is essentially obtained as the proportion of DNA fragments that belong to one of the binding

events (i.e., not correspond to background) but do not overlap positions of binding events (μ_g). As iterations progress in the EM algorithm, estimates of μ_g improve and number of such DNA fragments decreases. As a result, in the later iterations of EM algorithm, γ estimate becomes close to zero and $P(s|l;\mu_g,\gamma)$ converges to Uniform distribution.

We summarize the fragment generating process as follows:

1. Draw group index of the DNA fragment, $(Z_{i0}, Z_{i1}, Z_{i2}, \cdots, Z_{ig^*})$, from Multinomial$(1, (\pi_0, \pi_1, \pi_2, \cdots, \pi_{g^*}))$.

2. Draw library size, L_i, from known distribution $P(L)$.

3. Draw start position of the DNA fragment, S_i, conditional on Z_i and L_i:

   (a) If the DNA fragment belongs to g-th binding event ($Z_{ig} = 1, 1 \leq g \leq g^*$), draw start position of the fragment, S_i, from $P(S|L;\mu_g,\gamma)$.

   (b) If the DNA fragment is from background ($Z_{i0} = 1$), draw S_i from Uniform$(1-L_i+1, m)$.

2.2 Generative model for single-end tag (SET) data

In the SET data, one of two ends of each DNA fragment is randomly selected and sequenced. Hence, L_i for each fragment is not observable; however, positions and strands of the reads corresponding to the sequenced ends are known (denoted by R_i and D_i, respectively). We assume that D_i follows Bernoulli distribution with known parameter p_D.

Exploratory analysis indicates that these read distributions can be well approximated with Normal distribution. Specifically, we assume that

$$(R|Z=g, D=1; \mu_g, \delta, \sigma^2) \sim N(\mu_g - \delta, \sigma^2),$$

and

$$(R|Z=g, D=0; \mu_g, \delta, \sigma^2) \sim N(\mu_g + \delta, \sigma^2).$$

Note that δ corresponds to the half of the distance between modes of the binding event reads in forward and backward strands. We summarize the SET read generating process as follows:

1. Draw group index of the read, $(Z_{i0}, Z_{i1}, Z_{i2}, \cdots, Z_{ig^*})$, from Multinomial$(1, (\pi_0, \pi_1, \pi_2, \cdots, \pi_{g^*}))$.

2. Draw strand of the read, D_i, from Bernoulli(p_D).

3. Draw position of the read, R_i, conditional on Z_i and D_i:

   (a) If the read belongs to g-th binding event ($Z_{ig} = 1, 1 \leq g \leq g^*$) and it is in the forward strand ($D_i = 1$), draw position of the read, R_i, from Normal($\mu_g - \delta, \sigma^2$).

   (b) If the read belongs to g-th binding event ($Z_{ig} = 1, 1 \leq g \leq g^*$) and it is in the reverse strand ($D_i = 0$), draw position of the read, R_i, from Normal($\mu_g + \delta, \sigma^2$).

   (c) If the read is from background ($Z_{i0} = 1$) and it is in the forward strand ($D_i = 1$), draw position of the read, R_i, from Uniform($1 - \beta + 1, m$).

   (d) If the read is from background ($Z_{i0} = 1$) and it is in the reverse strand ($D_i = 0$), draw position of the read, R_i, from Uniform($1, m + \beta - 1$).

3 The dPeak Algorithm

We estimate parameters of the models for PET and SET data using the Expectation-Maximization (EM) algorithm [3]. We do not have explicit solutions in the M-step for the PET model. Maximization with respect to $(\mu_1, \mu_2, \cdots, \mu_{g^*})$ requires searching over g^*-dimensional space and $O(m^{g^*})$ operations, which is computationally prohibitive. In order to boost up computation and stabilize estimation, we employ the Expectation-Conditional-Maximization (ECM) algorithm [4]. The ECM algorithm requires only searching over one-dimensional space, $[1, m]$, for the maximization with respect to each μ_g while keeping the other parameters fixed. This reduces the computation time to $O(mg^*)$ operations. Our simulation studies show that this approach is computationally efficient and provides fast convergence with accurate and stable estimation (data not shown). We have explicit solutions in the M-step for the SET model.

Although the EM algorithm has desirable convergence properties, it does not guarantee convergence to the global maximum when there are multiple maxima. As a result, the final estimates depend upon the initial values [5, 6]. In order to address this issue, we consider the stochastic EM algorithm [7], which is a special case of Monte Carlo EM [5, 6], for the first half of iterations. The stochastic EM algorithm allows a chance of escaping from a current path of convergence to a local maximizer to other paths [5]. After certain number of iterations, we switch to the ordinary version of our EM algorithm because the stochastic EM is not desirable when the process is near to convergence to a suitable local maximizer [5].

In the EM implementation, non-identifiability due to overfitting (fitting too many components in the model) is problematic and should be avoided [5, 8]. We address this issue during the EM iterations as follows. If the distance between two binding events is shorter than the size of the binding site (defined by the length of the known or predicted consensus motif), we combine these two components and consider it as one component during the remaining iterations. For the σ^{70} application, we set this parameter to $20bp$ since σ^{70} binds to $-35bp$ and $-10bp$ from transcription start site. Moreover, if the strength of a binding event is too weak ($\pi_g < 0.01$), this component is also removed from further consideration in the remaining iterations.

3.1 The dPeak algorithm for PET data

Given the generative model for PET data described in Section 2.1, we have the following complete likelihood:

$$
\begin{aligned}
L_C = \quad & \prod_{i=1}^n P(L_i) \quad \left\{ \pi_0 \frac{1\left\{1 - L_i + 1 \le S_i \le m\right\}}{m + L_i - 1} \right\}^{Z_{i0}} \\
& \prod_{g=1}^{g*} \left\{ \pi_g \left[\frac{(1-\gamma)}{L_i} \right]^{1\{S_i \in B_g\}} \left[\frac{\gamma}{m-1} \right]^{1\{S_i \in C \backslash B_g\}} \right\}^{Z_{ig}}
\end{aligned}
$$

Let $\mathbf{S} = (S_1, S_2, \cdots, S_n)$, $\mathbf{L} = (L_1, L_2, \cdots, L_n)$, and $\mathbf{\Theta^{(t)}} = \left(\pi_0^{(t)}, \pi_1^{(t)}, \pi_2^{(t)}, \cdots, \pi_{g*}^{(t)}, \mu_1^{(t)}, \mu_2^{(t)}, \cdots, \mu_{g*}^{(t)}, \gamma^{(t)} \right)$. Then, the EM algorithm for the PET data is obtained as follows:

E-step:
For $g = 1, 2, \cdots, g^*$,

$$
\begin{aligned}
z_{ig}^{(t)} &= E\left(Z_{ig} | \mathbf{S}, \mathbf{L}, \mathbf{\Theta^{(t)}} \right) \\
&= \frac{\pi_g^{(t)}}{A} \left[\frac{(1-\gamma^{(t)})}{L_i} \right]^{1\left\{S_i \in B_g^{(t)}\right\}} \left[\frac{\gamma^{(t)}}{m-1} \right]^{1\left\{S_i \in C \backslash B_g^{(t)}\right\}},
\end{aligned}
$$

and for $g = 0$,

$$
\begin{aligned}
z_{i0}^{(t)} &= E\left(Z_{i0} | \mathbf{S}, \mathbf{L}, \mathbf{\Theta^{(t)}} \right) \\
&= \frac{\pi_0^{(t)}}{A(m + L_i - 1)},
\end{aligned}
$$

where A is an appropriate normalizing constant.

M-step:
For $g = 1, 2, \cdots, g^*$, we obtain

$$
\mu_g^{(t+1)} = argmax_{\mu_g} \sum_{i=1}^n z_{ig}^{(t)} \left[1\left\{S_i \in B_g\right\} \log \frac{(1-\gamma^{(t)})}{L_i} + 1\left\{S_i \in C \backslash B_g\right\} \log \frac{\gamma^{(t)}}{m-1} \right].
$$

and

$$
\pi_g^{(t+1)} = \frac{1}{n} \sum_{i=1}^n z_{ig}^{(t)}.
$$

Similarly,

$$\pi_0^{(t+1)} = \frac{1}{n}\sum_{i=1}^{n} z_{i0}^{(t)}.$$

Moreover,

$$\gamma^{(t+1)} = \frac{1}{n}\sum_{i=1}^{n}\sum_{g=1}^{g^*} z_{ig}^{(t)} 1\left\{S_i \in C \backslash B_g^{(t+1)}\right\}.$$

This algorithm has the following intuitive interpretation. In the E step, each fragment is allocated to a binding event or background component based on whether or not the fragment overlaps the actual binding events. When the fragment overlaps with more than one binding events, it is assigned to each of these events in a fractional manner. The fractions are proportional to relative strengths of the binding events (π_g). In the M step, location of each binding event (μ_g) is essentially updated to the position with the largest number of aligning fragments. In this step, fragments with shorter library size (L_i) have more voting power. This is intuitive from the experimental procedure point of view because it is easier to identify the actual position of a binding event with shorter fragments.

3.2 The dPeak algorithm for SET data

Given the generative model for SET data described in Section 2.2, we have the following complete likelihood:

$$\begin{aligned} L_C = \quad & \prod_{i=1}^{n} \quad \{\pi_0 \left[p_D \frac{1\{1-\beta+1 \le R_i \le m\}}{m+\beta-1}\right]^{1\{D_i=1\}} \\ & \quad \left[(1-p_D)\frac{1\{1 \le R_i \le m+\beta-1\}}{m+\beta-1}\right]^{1\{D_i=0\}} \}^{Z_{i0}} \\ & \prod_{g=1}^{g*} \quad \{\pi_g \frac{1}{\sqrt{2\pi(\sigma^2)}} \left[p_D \exp\left\{-\frac{1}{2(\sigma^2)}(R_i-(\mu_g-\delta))^2\right\}\right]^{1\{D_i=1\}} \\ & \quad \left[(1-p_D)\exp\left\{-\frac{1}{2(\sigma^2)}(R_i-(\mu_g+\delta))^2\right\}\right]^{1\{D_i=0\}} \}^{Z_{ig}} \end{aligned}$$

Let $\mathbf{R} = (R_1, R_2, \cdots, R_n)$, $\mathbf{D} = (D_1, D_2, \cdots, D_n)$, and

$\boldsymbol{\Theta}^{(\mathbf{t})} = \left(\pi_0^{(t)}, \pi_1^{(t)}, \pi_2^{(t)}, \cdots, \pi_{g*}^{(t)}, \mu_1^{(t)}, \mu_2^{(t)}, \cdots, \mu_{g*}^{(t)}, \delta^{(t)}, (\sigma^2)^{(t)}\right)$. Then, the EM algorithm for the SET data is obtained as follows:

E-step:
For $g = 1, 2, \cdots, g^*$,

$$\begin{aligned} z_{ig}^{(t)} &= E(Z_{ig}|\mathbf{R}, \mathbf{D}, \boldsymbol{\Theta}^{(\mathbf{t})}) \\ &= \frac{\pi_g^{(t)}}{A\sqrt{2\pi(\sigma^2)^{(t)}}} \left[p_D \exp\left\{-\frac{1}{2(\sigma^2)^{(t)}}(R_i - (\mu_g^{(t)} - \delta^{(t)}))^2\right\}\right]^{1\{D_i=1\}} \\ &\qquad \left[(1-p_D) \exp\left\{-\frac{1}{2(\sigma^2)^{(t)}}(R_i - (\mu_g^{(t)} + \delta^{(t)}))^2\right\}\right]^{1\{D_i=0\}}, \end{aligned}$$

and for $g = 0$,

$$\begin{aligned} z_{i0}^{(t)} &= E\left(Z_{i0}|\mathbf{R}, \mathbf{D}, \boldsymbol{\Theta}^{(\mathbf{t})}\right) \\ &= \frac{\pi_0^{(t)} p_D^{1\{D_i=1\}}(1-p_D)^{1\{D_i=0\}}}{A(m+\beta-1)}, \end{aligned}$$

where A is an appropriate normalizing constant.

M-step:
For $g = 1, 2, \cdots, g^*$, we obtain

$$\mu_g^{(t+1)} = \frac{1}{\sum_{i=1}^n z_{ig}^{(t)}} \sum_{i=1}^n z_{ig}^{(t)} \left[(R_i + \delta^{(t)})1\{D_i = 1\} + (R_i - \delta^{(t)})1\{D_i = 0\}\right],$$

and

$$\pi_g^{(t+1)} = \frac{1}{n}\sum_{i=1}^n z_{ig}^{(t)}.$$

Similarly,

$$\pi_0^{(t+1)} = \frac{1}{n}\sum_{i=1}^n z_{i0}^{(t)}.$$

Moreover,

$$\delta^{(t+1)} = \frac{1}{n}\sum_{i=1}^n \sum_{g=1}^{g^*} z_{ig}^{(t)} \left[(\mu_g^{(t+1)} - R_i)1\{D_i = 1\} + (R_i - \mu_g^{(t+1)})1\{D_i = 0\}\right],$$

and

$$(\sigma^2)^{(t+1)} = \frac{1}{n}\sum_{i=1}^{n}\sum_{g=1}^{g^*} z_{ig}^{(t)} \quad [(R_i - (\mu_g^{(t+1)} - \delta^{(t+1)}))^2 1\{D_i = 1\} + (R_i - (\mu_g^{(t+1)} + \delta^{(t+1)}))^2 1\{D_i = 0\}].$$

This algorithm has the following intuitive interpretation. In the E step, each read is allocated to a binding event or background component based on the distance between the binding events and the read shifted by δ towards its $3'$ direction. Both the peak shape (p_D, δ, and σ^2) and the relative strengths of the binding events (π_g) are considered in this allocation. In the M step, location of each binding event (μ_g) is updated to the averaged position of reads corresponding to the binding event, after reads are shifted by δ towards their $3'$ direction. One peak shape is estimated for each candidate region through δ and σ^2. Optimal shift of reads from their corresponding binding events, δ, is updated to the averaged distance between the location of each binding event and the positions of reads corresponding to this binding event, averaged over binding events in the region. Dispersion of the reads around their corresponding binding events, σ^2, is updated to the variance of the position of reads corresponding to the binding event around location of each binding event (μ_g), after reads are shifted by δ to their $3'$ direction, averaged over binding events in the region.

3.3 Model selection

In practice, determining the optimal number of binding events, g^*, in each candidate region can be cast as a model selection problem. Model selection based on the Bayesian Information Criterion (BIC) [9] is a popular choice in mixture modeling and has shown superior performance in diverse applications [10, 11]. Therefore, for pre-specified g^{max}, we fit models for each of $g^* = 1, 2, \cdots, g^{max}$ binding event components and choose the model with the BIC value corresponding to the first local minimum, as the final model.

Choice of g^{max} is an important issue in model selection. g^{max} should be large enough so that all binding events in each candidate region can be considered. On the other hand, setting g^{max} larger than necessary should also be avoided in order to prevent choosing a model due to ill-conditioning rather than a genuine indication of a better model [10, 11]. For appropriate choice of g^{max} in the current application, we checked the number of known binding events in each candidate region of σ^{70} data from the RegulonDB database [12] (`http://regulondb.ccg.unam.mx`) and found

that 92% of the peaks have either one or two binding sites within the peak region. Based on this exploratory analysis, we set $g^{max} = 5$ as the default value and use it for all the analysis described in the manuscript. For other applications, appropriate choice of g^{max} might depend on the protein type and experimental conditions.

4 Estimation of the Optimal Shift in the dPeak Algorithm

Figure S2A displays the density of library size in the σ^{70} PET ChIP-Seq data. The corresponding mean and standard deviation are $192.01bp$ and $26.90bp$, respectively. Figure S2B shows the estimated density of 2δ in the σ^{70} quasi-SET ChIP-Seq data, where δ is the half of the distance between modes of forward and reverse strand reads belonging to each binding event in the candidate region. Mean and standard deviation of 2δ are $187.36bp$ and $9.04bp$, respectively. Figure S2C depicts the scatter plot of library size vs. estimated 2δ and it indicates that, overall, we have larger 2δ estimates for the candidate regions with larger average library sizes. We observe the same pattern in Figure S2D, which displays a similar plot for PET and SET simulation data.

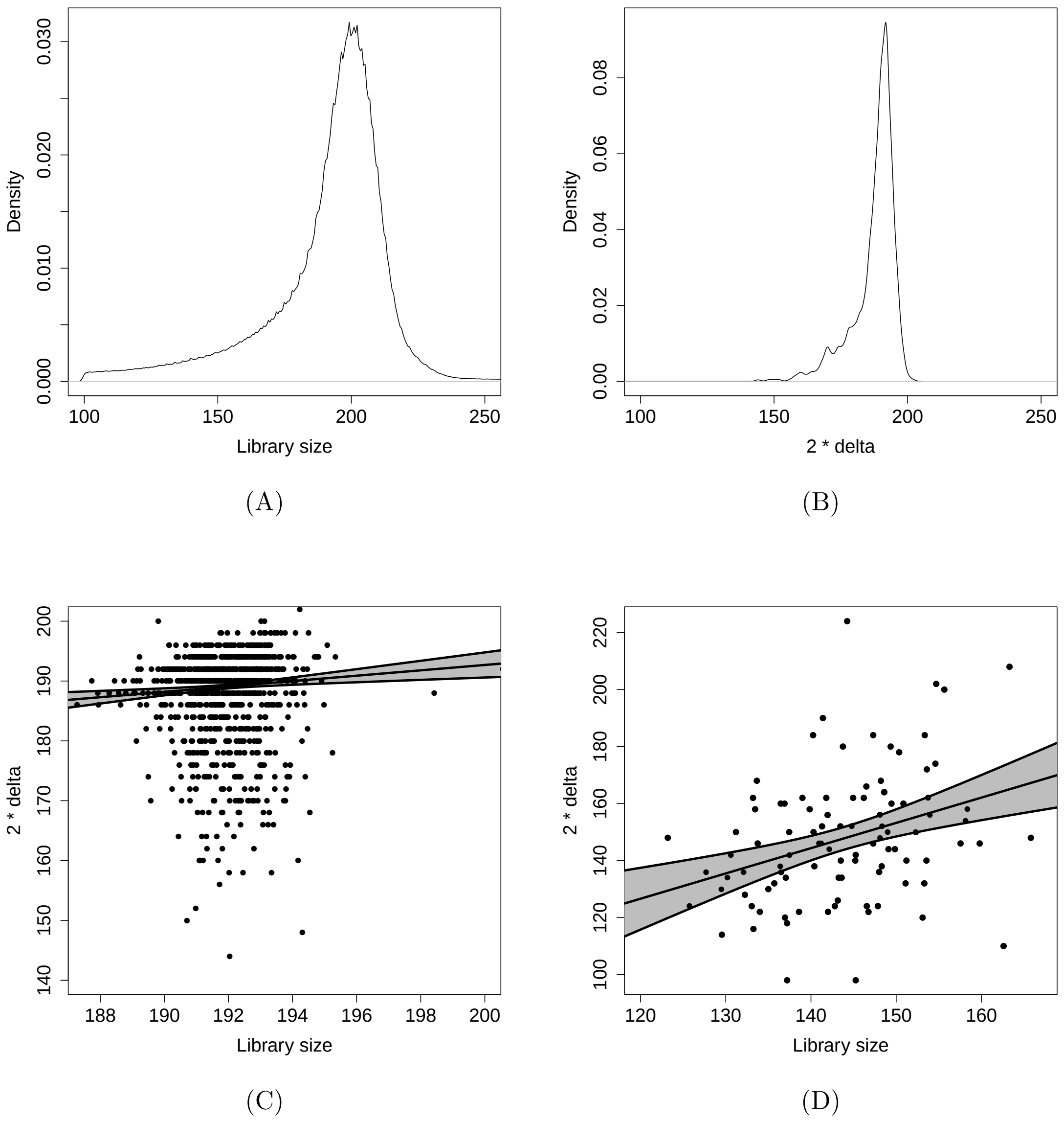

Figure S2: (A) Empirical density of the library size in the σ^{70} PET ChIP-Seq data. (B) Density of estimated 2δ in the σ^{70} quasi-SET ChIP-Seq data. (C) Scatter plot of library size vs. estimated 2δ in the σ^{70} PET and quasi-SET ChIP-Seq data. (D) Scatter plot of library size vs. estimated 2δ in PET and SET simulation data. In (C) and (D), the solid line and shades indicate a robust linear model (RLM) fit and the corresponding confidence intervals, respectively.

5 Diagnostics of the dPeak Model

Figures S3A, B display the goodness of fit (GOF) plots of the analysis displayed in Figure 4C for the PET and quasi-SET ChIP-Seq data, respectively. GOF plots compare the empirical distribution of the read positions with that obtained by simulating from estimated model parameters. These GOF plots are representative of the GOF plots for other candidate regions and they indicate that the dPeak models fit the data well.

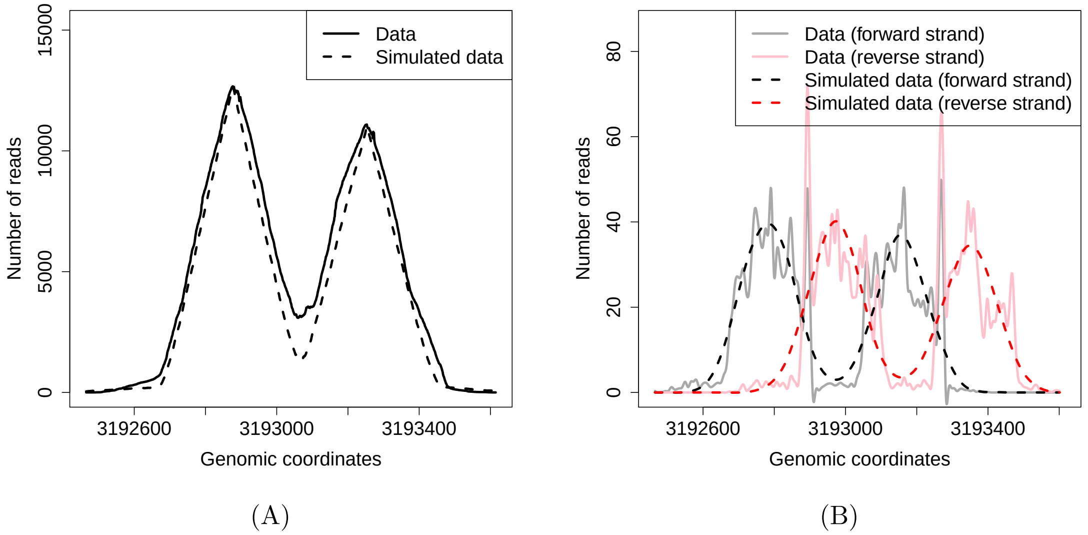

Figure S3: Goodness of fit (GOF) plot of the analysis displayed in Figure 4C, for the PET (A) and the quasi-SET (B) ChIP-Seq data, respectively.

6 Comparison of Deconvolution Algorithms

	dPeak	PICS	GPS/GEM[a]
Support PET data	Yes	No	No
Support SET data	Yes	Yes	Yes
Consider non-specific binding	Yes	No	No
Consider local shift of reads	Yes	Yes	No
Construct candidate regions	Utilize both ChIP and control samples	Utilize only ChIP sample	Utilize only ChIP sample
Peak shape estimation	Parametric[b]	Parametric[c]	Nonparametric[d]
Normalization	Normalization using non-specific binding	Normalization by sequencing depth	Regression approach
Merging	No	Yes	No
Filtering	Yes	Yes	Yes
Software interface	R and Galaxy	R	Java
Support parallel computing	Yes	Yes	Yes
Supported aligned read file formats	BED, Eland result, Eland extended, Eland export, Bowtie, SAM	BED, Eland result, Eland export, Bowtie, BAM, SOAP, MAQ[e]	BED, SAM, Bowtie, ELAND, NovoAlign

Table S3: Comparison of deconvolution algorithms. (a) GEM is a modified and extended version of GPS and it additionally incorporates sequence information to improve identification of binding events. (b) Use Uniform distribution for PET data and normal distribution for SET data, respectively, for binding event components, in addition to Uniform distribution for the background component. (c) Use t-distribution with degree of freedom 4, for binding event components. (d) Requires users provide initial peak shape. (e) File formats supported by `ShortRead` package.

7 Effects of the Merging Step in PICS for Closely Spaced Binding Events

PICS [13] generates initial predictions for locations of protein binding events and then merges initial predictions that have overlapping "binding event neighborhoods". A binding event neighborhood is defined as the predicted location of a binding event extended by three standard errors of the shift parameter estimate to both sides. In order to evaluate the effect of merging on PICS binding event predictions, we regenerated results in Figures 2A, B without the merging step for PICS. Figures S4A, B show that PICS without merging step performs comparable to dPeak for SET ChIP-Seq data and the merging step of PICS results in loss of resolution for closely spaced binding events. Although it might be possible to tune the merging step, PICS currently does not provide this functionality.

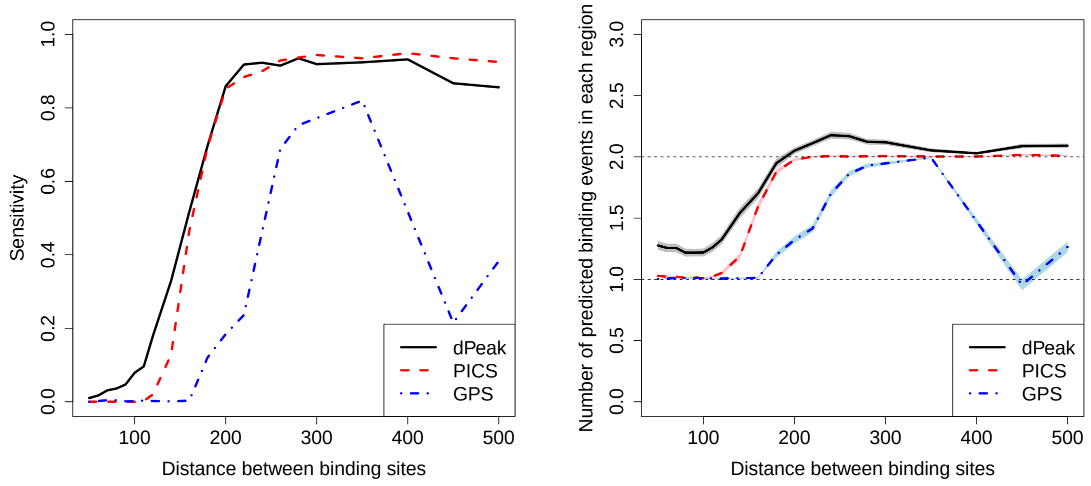

(A) (B)

Figure S4: Sensitivity (A) and positive predictive value (B) comparisons of high resolution binding site identification methods in computational experiments designed for the GPS algorithm. In these evaluations, the merging step is skipped in PICS as opposed to the evaluations obtained by default parameters of PICS in Figures 2A, B of the main text.

8 Peak Shape Estimation of GPS for Closely Spaced Binding Events

Figure S5A displays the peak shape estimated by the GPS algorithm [14] for synthetic ChIP-Seq data when there is only one binding event in each candidate region. It depicts density of forward strand reads with respect to the distance from the location of binding event (corresponding to zero in the x axis). This same peak shape is used genome-wide for modeling of reads in both forward and reverse strands. When there is single binding event, peak shape is correctly estimated as uni-modal. Figure S5B displays the peak shape when the distance between two binding sites in each candidate region is set to $450bp$. The peak shape is still correctly estimated as uni-modal and it looks similar to the peak shape estimated for single binding events. Moreover, in these two cases, the estimated peak shapes are similar to their initial shapes. Figure S5C shows the estimated peak shape when the distance between two binding sites in each candidate region is set to $140bp$. In this case, both of the two closely spaced binding events affect peak shape estimation of the GPS algorithm. As a result, the peak shape is estimated as bi-modal, which in turn leads to predicting the two binding events as a single event after a few rounds of the GPS iterations. We note that this problem typically occurs for nonparametric mixture models when the distances between mixture components are relatively short compared to the bandwidth.

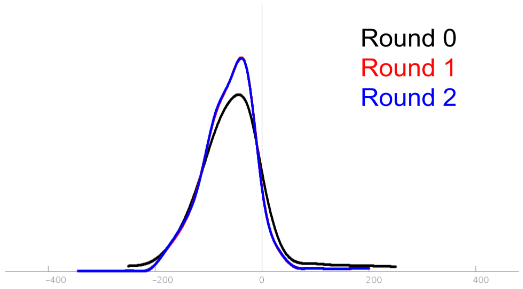

(A)

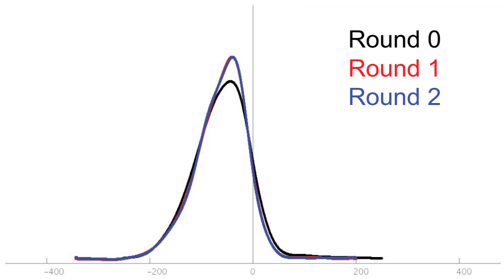

(B)

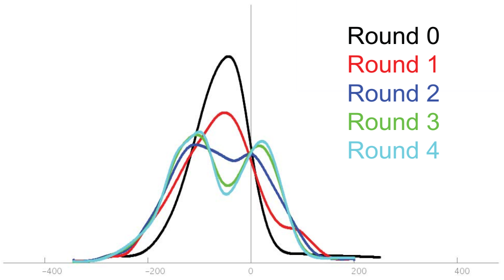

(C)

Figure S5: Peak shapes estimated by the GPS algorithm for synthetic ChIP-Seq data: (A) when there is a single binding event; (B, C) when the distance between joint binding events is set to $450bp$ (B) and $140bp$ (C). "Round" denotes the iteration number in the algorithm and "Round 0" depicts the initiation.

9 Evaluations on Synthetic Data from [14] with a Single Binding Event

# of predicted events	0	1	> 1	Average # of events
dPeak	0%	86 %	14%	1.16 (0.42)
PICS	1%	97%	2%	1.02 (0.16)
GPS	82%	6%	12%	2.72 (1.69)

Table S4: Prediction accuracy for 20,000 candidate regions with single binding event. Columns 2-4 report percentages of candidate regions with various numbers of predicted binding events. Column 5 reports the average number of binding events across regions with at least one predicted binding event.

10 Evaluations on Simulation Data with a Single Binding Event

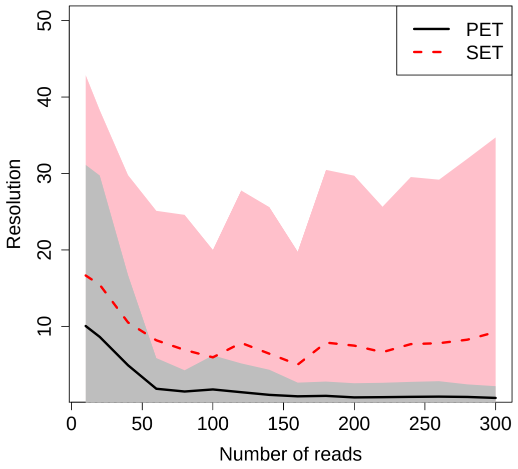

Figure S6: Resolution of predictions as a function of number of DNA fragments in PET and SET simulated data with a single binding event. Resolution is defined as the absolute distance between the predicted and true binding event positions. Black solid and red dotted curves indicate averaged resolutions for each number of DNA fragments in PET and SET data, respectively. Gray and pink shades indicate their confidence intervals in PET and SET data, respectively.

11 Evaluations on Simulation Data based on Different Data Generation Process

When comparing PET and SET data with simulations (Figures 2C, D and Figure S6), we first generated PET data and then obtained corresponding SET data by randomly sampling one of two ends of each resulting DNA fragment. Although such a data generation process closely mimics the process for generating real SET ChIP-Seq data, dPeak model for SET ChIP-Seq data capitulates this process by a Normal approximation of the density of each of forward and reverse strand reads. In order to assure that our evaluation using random sampling does not give unwarranted advantages to PET data, we generated SET data with read positions directly originating from Normal distribution and repeated the analysis in Figures 2C, D. Figures S7A, B, C confirm that the comparisons between PET and SET data remain the same regardless of how SET data is simulated.

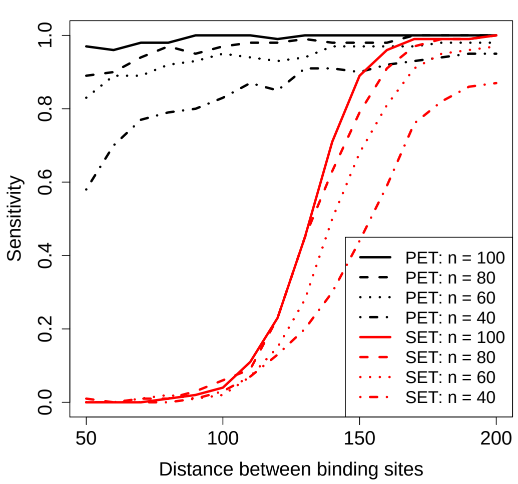

(A)

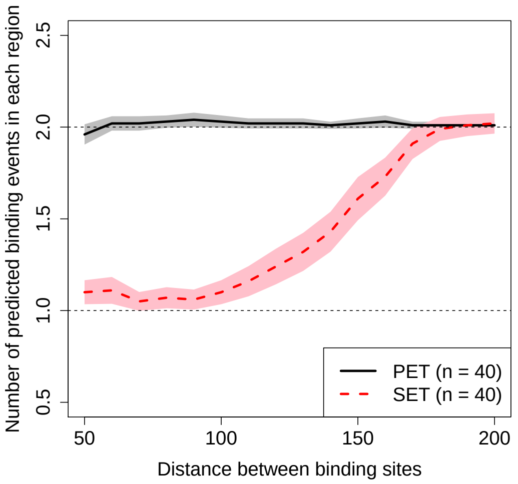

(B)

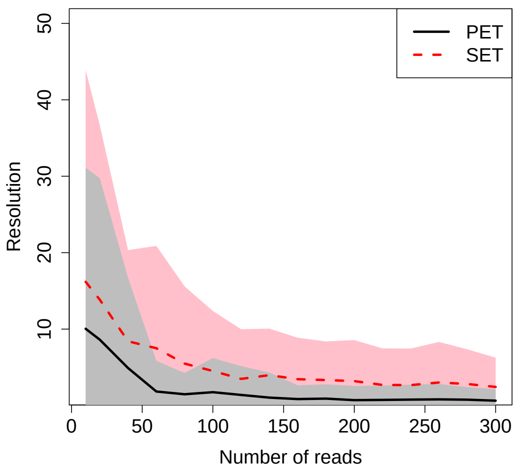

(C)

Figure S7: Sensitivity (A), positive predictive value (B), and resolution (C) comparisons of dPeak performance on PET vs. SET data when SET read density is directly generated from Normal distribution. n indicates number of reads corresponding to each binding event and $n/2$ DNA fragments are used for PET data to match the number of reads between PET and SET data. Shaded areas around each line indicate confidence intervals. Results are similar to those in Figures 2C, D, and Figure S6, where SET data is generated by random sampling of one of the two ends from each DNA fragment in PET data.

12 Analytical Calculations for Invasion and Truncation

Consider a region with two closely located binding events. Processing of DNA fragments generated from this region will lead to classification of the fragments in one of the following four categories:

Category I: Fragments overlapping a single true binding event.

Category II: Fragments overlapping both binding events.

Category III: Fragments overlapping only the false binding event.

Category IV: Fragments not overlapping any binding events.

Only fragments in category I are truly informative. Fragments in category II are less informative than fragments in category I. They could potentially contribute to both binding events, possibly through proportional allocation based on relative distances from each binding event. However, ambiguity in prediction increases as the number of fragments in category II increases. Fragments in category III introduce noise to binding event estimation since they are associated with the wrong binding event. Fragments in category IV are uninformative. In summary, invasion refers to increased number of category II fragments in SET data compared to PET data and truncation refers to increased number of category III and IV fragments in SET data compared to PET data.

Table S5 displays the number of fragments in each category from one simulated dataset where we set the distance between the two binding events as $50bp$. Average library size is $139bp$ in the PET data. The estimated library size used with SET analysis are reported in parentheses in the first column. In the corresponding SET data, even when extension is relatively accurate (extension = $150bp$), numbers of fragments in categories II to IV increase significantly compared to PET data. When the library size is under-estimated as $100bp$, we have significantly more fragments in categories III and IV (truncation; Figure S8B). In contrast, when it is over-estimated as $200bp$, we have significantly more fragments in category II (invasion; Figure S8A).

We used the dPeak generative model and calculated the probability of invasion and truncation (Figure S8) as follows. As in the previous sections, let S and L be start position of DNA fragment and its length, respectively, in PET ChIP-Seq data. Let l^* denote the fixed library size used in the analysis of SET ChIP-Seq data. Z indicates group index of the DNA fragment where $Z = 1$ and $Z = 2$ indicates correspondence to the first and second binding events, respectively. Let μ_1 and μ_2 be

Category	I	II	III	IV
	Informative	Less informative		
	Overlapping only true binding events	Overlapping both binding events	Overlapping only false binding event	Not overlapping any binding event
PET	225	375	0	0
SET (150)	174	391	19	16
SET (100)	232	215	89	64
SET (200)	133	461	3	3

Table S5: Classification of 600 DNA fragments from one simulated dataset with two binding events separated by $50bp$.

positions of first and second binding events, respectively, and assume that $\mu_1 < \mu_2$. Probability of invasion (Figure S8A) is obtained as:

$$\begin{aligned} P(Invasion) &= E_L[P(S < \mu_1 < S + L < \mu_2 < S + l^*|Z = 1, L)] \\ &= \sum_{L=l} P(L = l)P(S < \mu_1 < S + l < \mu_2 < S + l^*|Z = 1, L = l) \\ &= \sum_{L=l} P(L = l)min\left\{l, \mu_2 - \mu_1, l^* - l, l^* - (\mu_2 - \mu_1)\right\}/l. \end{aligned}$$

As illustrated in Figure S8B, for truncation, we consider the case that the original DNA fragment covers both binding events in PET data. The corresponding probability can be calculated by defining the truncation event with the use of the Z variable:

$$\begin{aligned} P(Truncation) &= E_L[P(S + l^* < \mu_2, S < \mu_1 < \mu_2 < S + L|Z = 2, L)] \\ &= \sum_{L=l} P(L = l)P(S + l^* < \mu_2, S < \mu_1 < \mu_2 < S + l|Z = 2, L = l) \\ &= \sum_{L=l} P(L = l)min\left\{l - l^*, l - (\mu_2 - \mu_1)\right\}/l. \end{aligned}$$

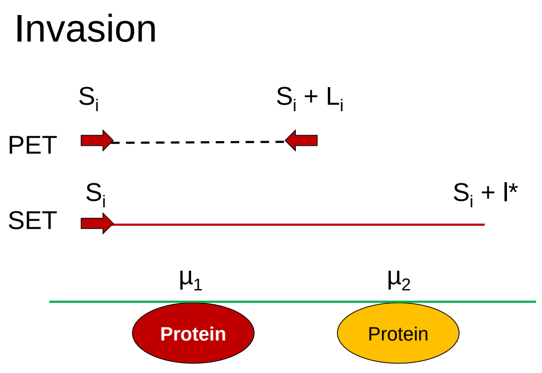

(A)

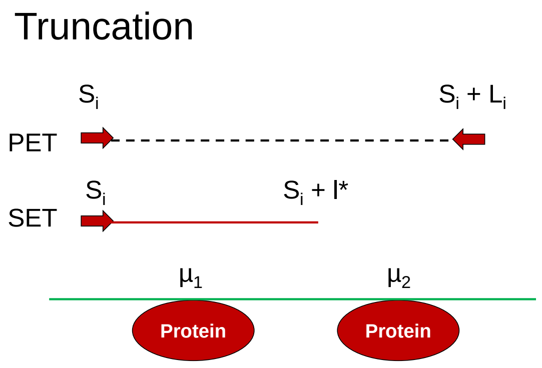

(B)

Figure S8: Concepts of *invasion* (A) and *truncation* (B). In each diagram, the first and second lines indicate PET and SET ChIP-Seq data, respectively. Red horizontal line depicts estimated library size in the SET data. Red circles denote the protein binding event that the read corresponds to.

13 Evaluations on σ^{70} PET and SET ChIP-Seq Data Using RegulonDB and Experimentally Validated Sites as a Gold Standard

We compared performances of deconvolution algorithms dPeak, PICS, GPS, and GEM using σ^{70} PET and quasi-SET ChIP-Seq data by considering RegulonDB annotated binding sites as a gold standard. We assessed sensitivity of each algorithm using the set of candidate regions with at least two annotated binding sites and evaluated resolution using the candidate regions with exactly one annotated binding site.

Table S6 and Figures S9A, B show that dPeak using PET ChIP-Seq data provides significantly higher sensitivity and resolution than SET ChIP-Seq data regardless of the deconvolution algorithm used. GPS performs the worst and its poor performance had recently motivated the development of GEM [15]. Overall, dPeak and GEM perform similarly and both are slightly better than PICS with SET data in terms of sensitivity.

We also compared deconvolution algorithms using our small set of experimentally validated binding sites as a gold standard. This comparison (Figure S9C) further confirmed our conclusions from the RegulonDB-based comparisons. The differences in resolution between dPeak using PET ChIP-Seq data and each of the deconvolution algorithms using SET ChIP-Seq data are statistically significant with p-values $<$ 0.01.

	dPeak (PET)	dPeak (SET)	PICS	GPS	GEM
$+O_2$	0.66	0.47	0.39	0.20	0.43
$-O_2$	0.64	0.43	0.41	0.10	0.47

Table S6: Sensitivity comparisons across regions with at least two annotated binding events for σ^{70} PET and quasi-SET ChIP-Seq data in aerobic and anaerobic conditions. RegulonDB annotated binding sites are used as a gold standard. A gold standard binding event is marked as identified if the distance between the prediction and the RegulonDB reported location is less than $30bp$ (overall conclusions remained the same with other distances).

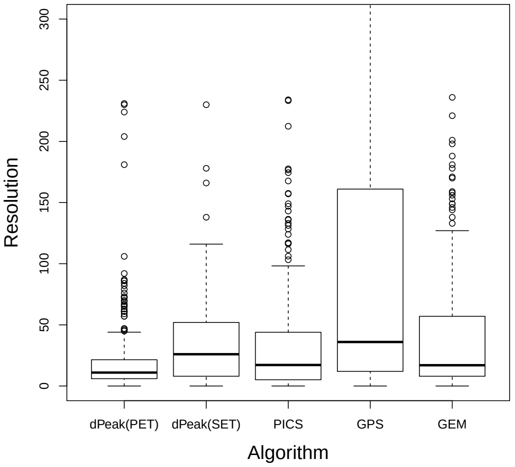

(A)

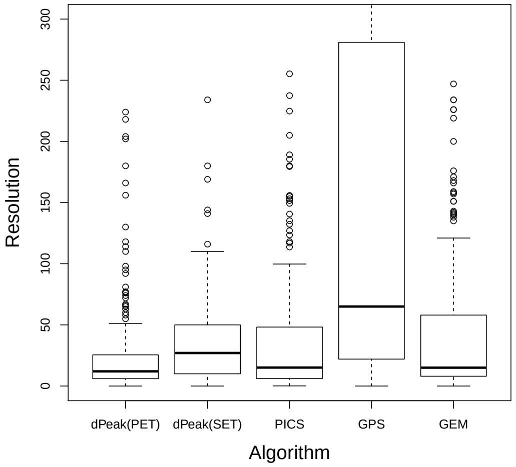

(B)

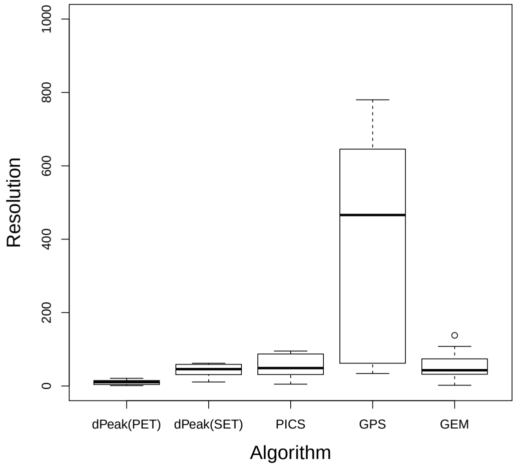

(C)

Figure S9: Resolutions of predictions for the regions with a single annotated binding event for σ^{70} PET and quasi-SET ChIP-Seq data in aerobic (A) and anaerobic (B) conditions when RegulonDB annotated binding sites are used as a gold standard. (C) Resolutions of predictions for σ^{70} PET and quasi-SET ChIP-Seq data using experimentally validated binding sites as a gold standard.

14 Experimental Validation of dPeak Predictions from σ^{70} PET ChIP-Seq Data

Name	DNA sequence
yejGP1	GGACGATTGAGAGTTGTAATG
yejGP2	CCTCTATGGCTCTGATTTAAG
sprP1	GTTTGTTTTCCCTTGAAGTCC
sprP2	CCAAATCTGTGGACTAACGCA
dcuAP1	GCATATTAGCCTTCCTTGTT
dcuAP2	CCCTGTACGATTACTGTTCG
serCP1	TTGAAGATTTGAGCCATTTCC
aroLP1	AAAGAGGTTGTGTCATCGTG
aroLP2	GCGATCATACCATCAAACTAG
hybOP1	CAATAATGCGATCGATGCGCC
ybgIP1	CGTTAATCAGTTGTTCCAGT
ptsGP1	TCCTGAGTATGGGTGCTTT

Table S7: Primers.

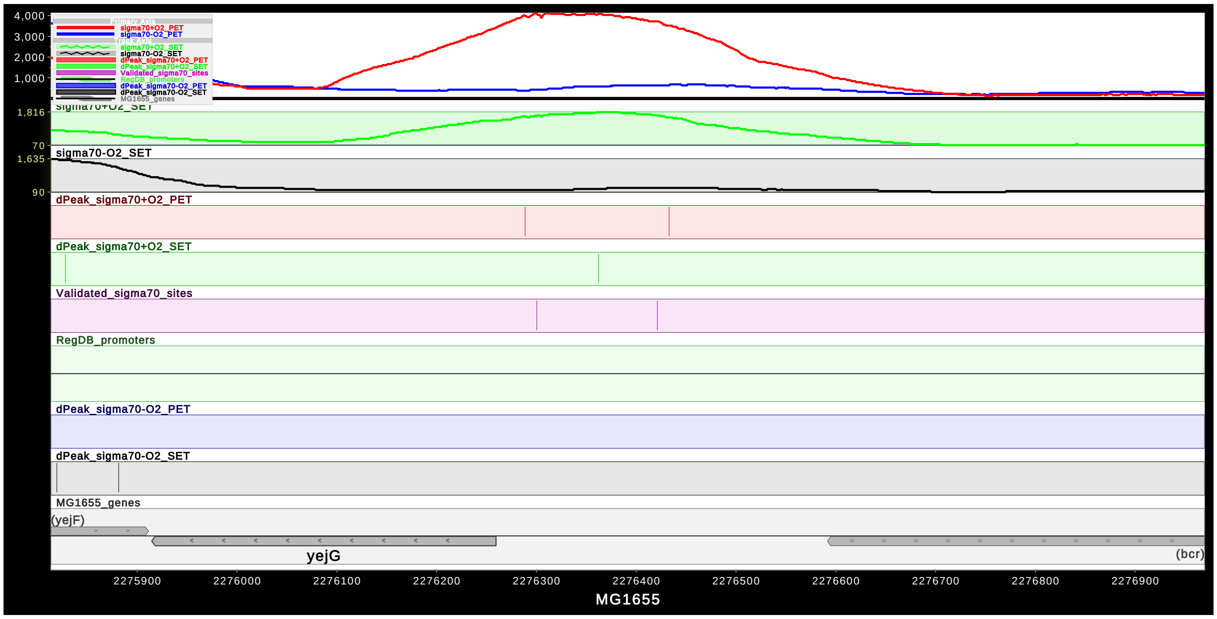

(A)

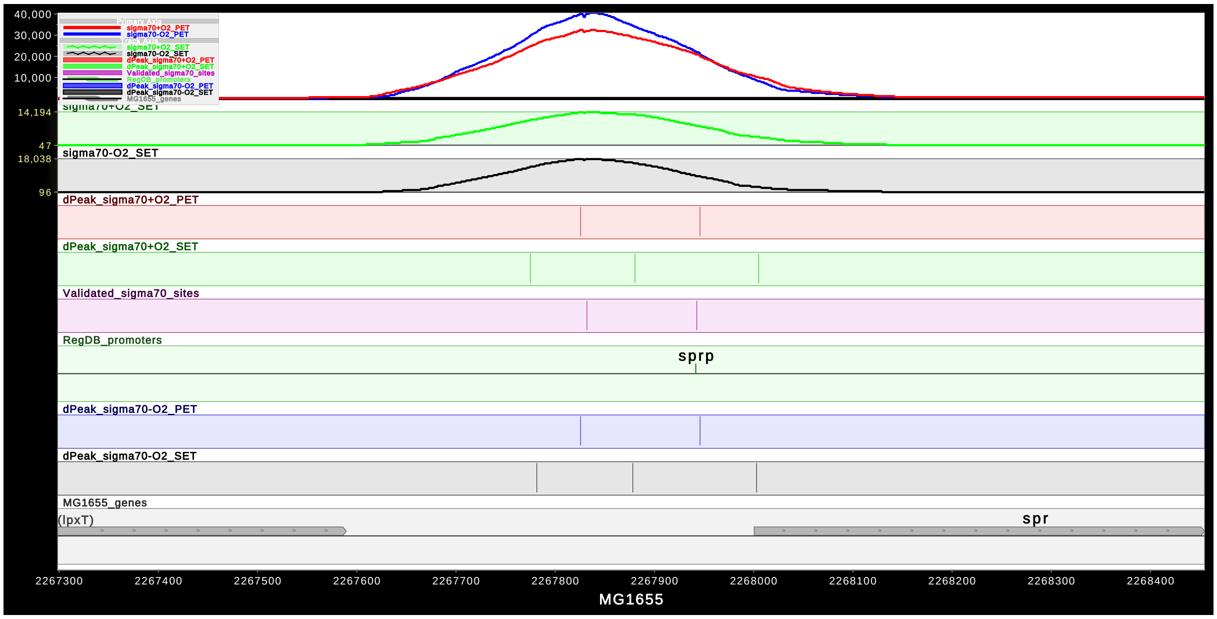

(B)

Figure S10: MochiView genome browser [16] screenshots of promoter regions of *yejG* (A) and *spr* (B) genes.

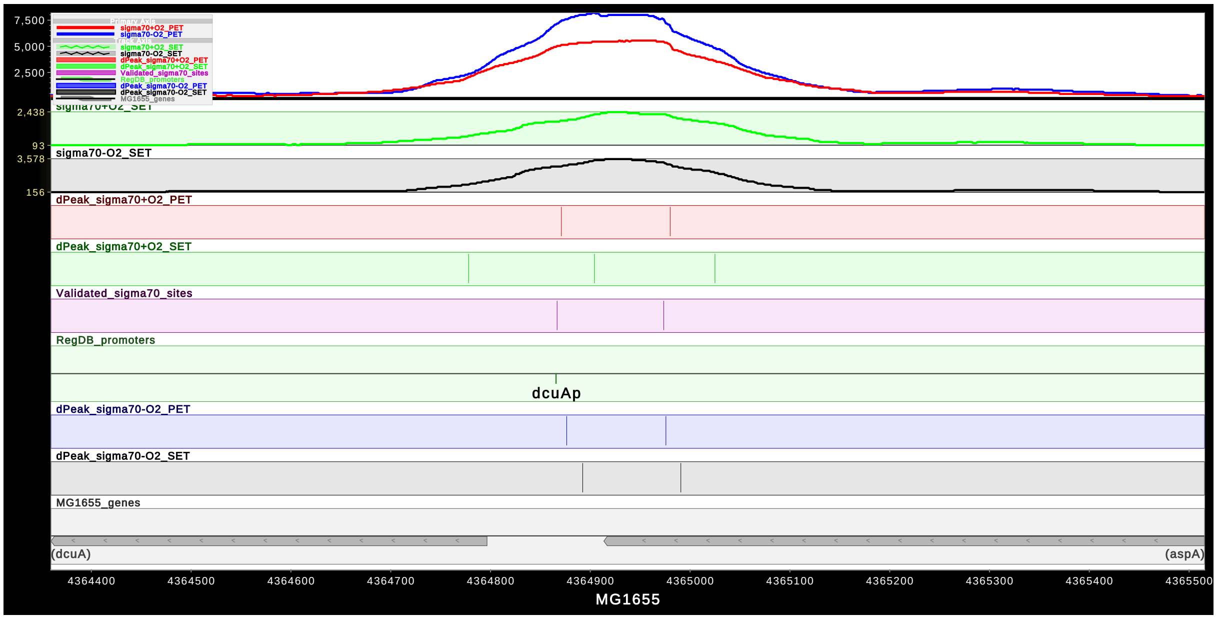

(A)

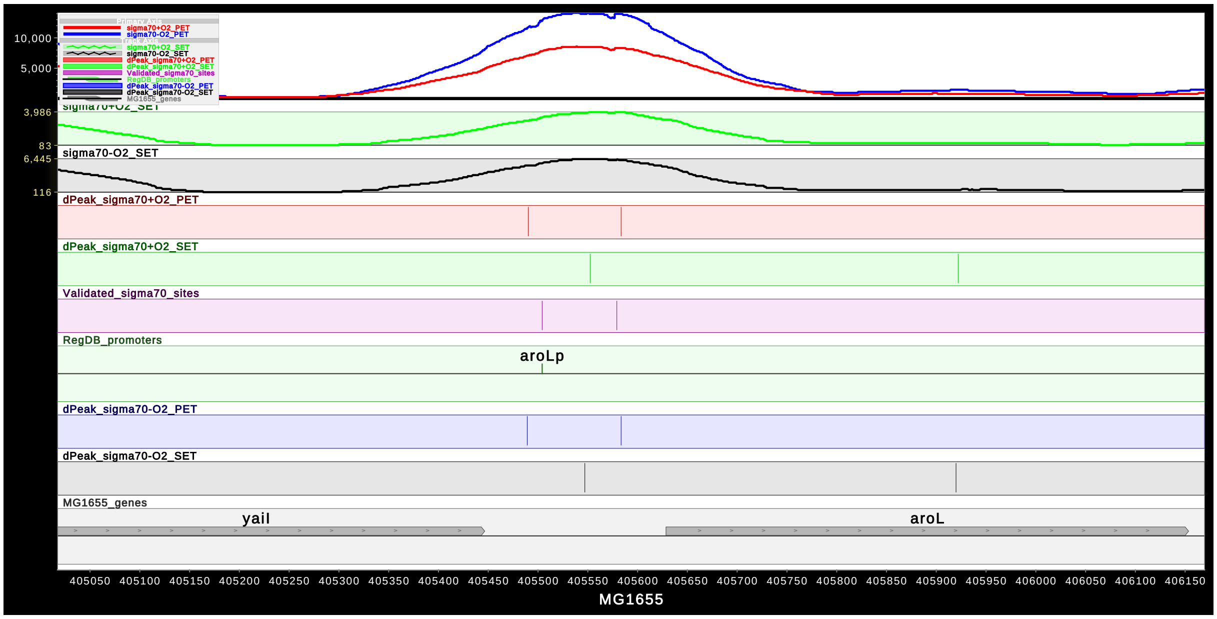

(B)

Figure S11: MochiView genome browser [16] screenshots of promoter regions of *dcuA* (A) and *aroL* (B) genes.

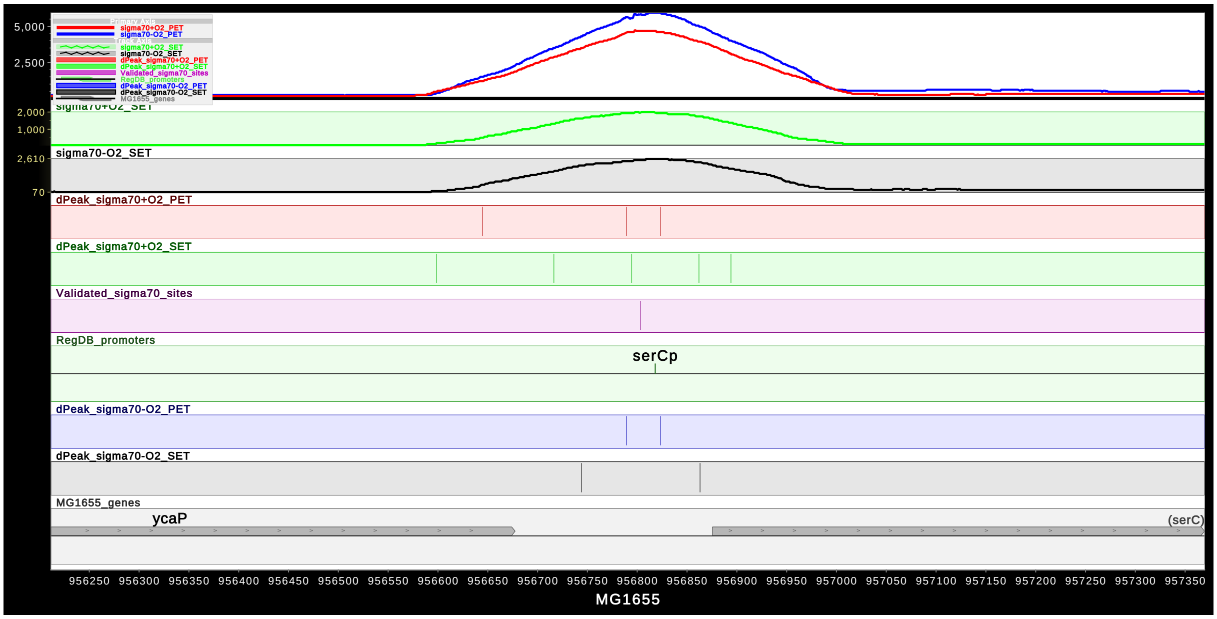

(A)

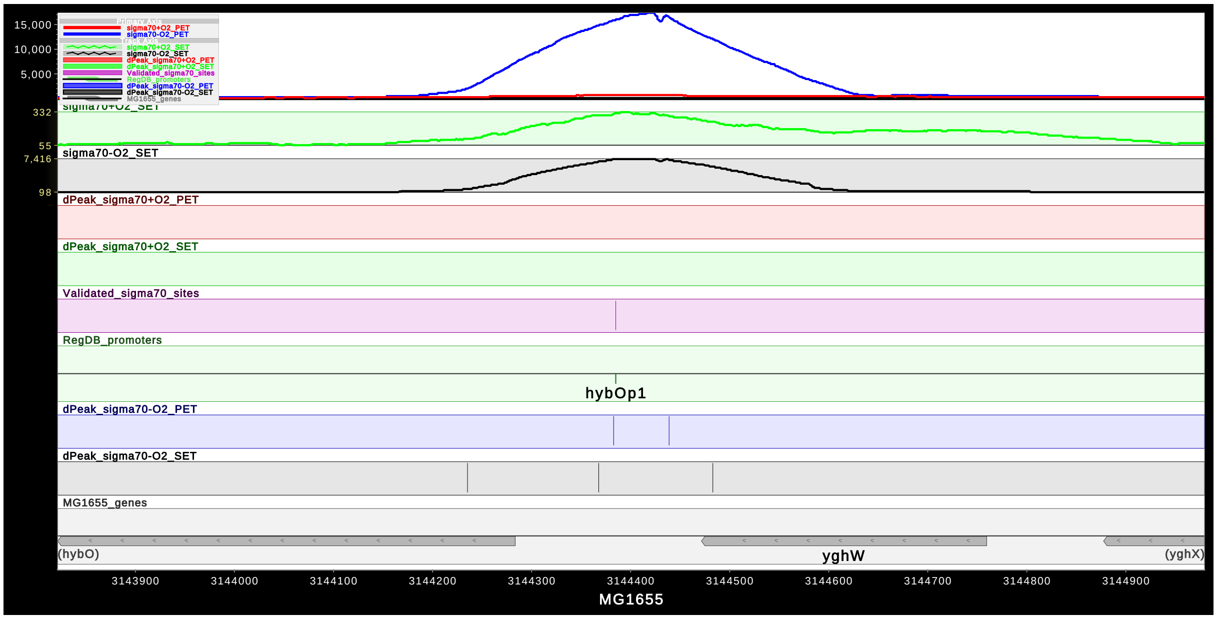

(B)

Figure S12: MochiView genome browser [16] screenshots of promoter regions of *serC* (A) and *hybO* (B) genes.

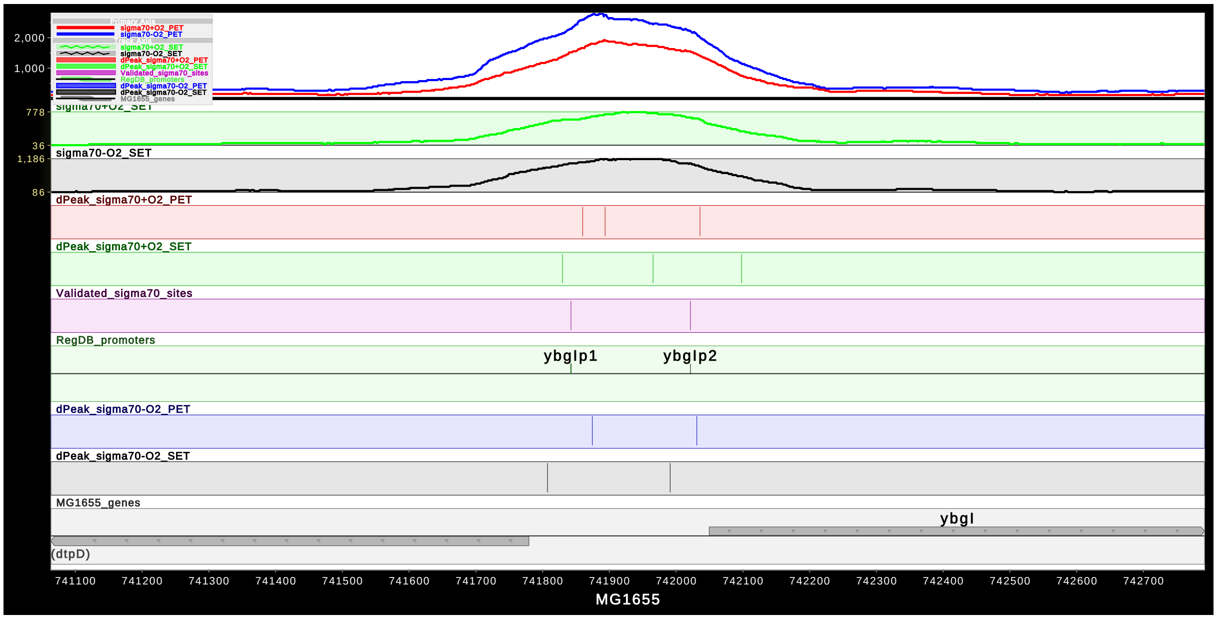

(A)

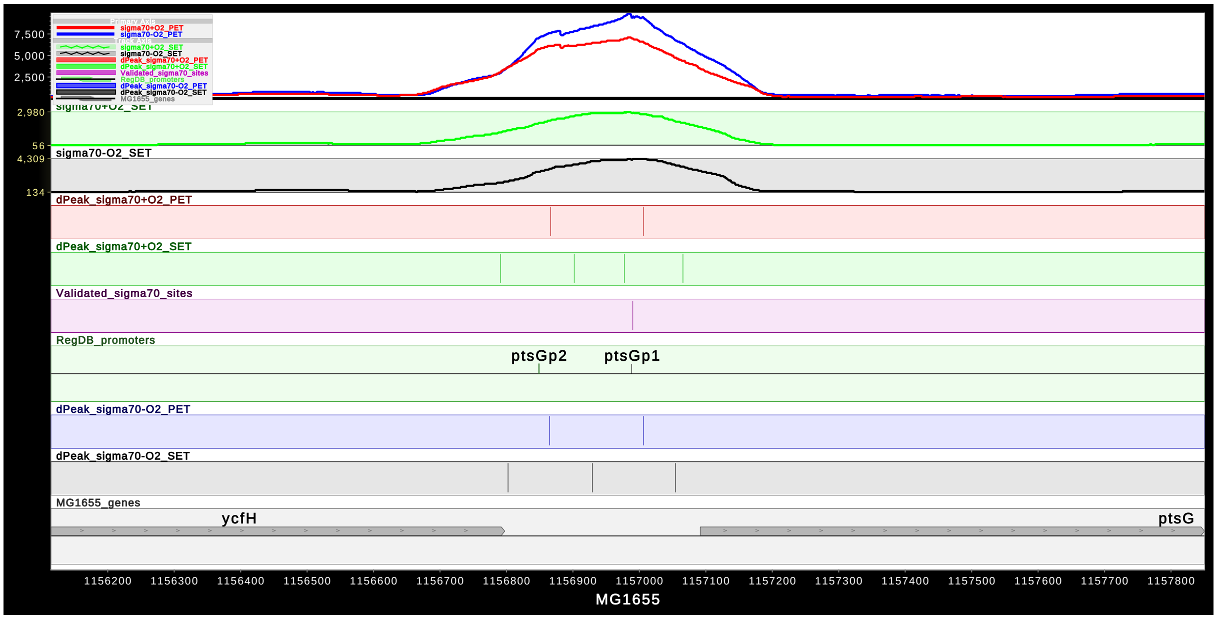

(B)

Figure S13: MochiView genome browser [16] screenshots of promoter regions of *ybgI* (A) and *ptsG* (B) genes.

15 Differential Occupancy of Closely Located Binding Sites between Aerobic and Anaerobic Conditions in *E. coli* σ^{70} PET ChIP-Seq Data

Figure 5C elucidates the merit of high resolution analysis in the studies of differential occupancy. However, if there is no occupancy in one condition at all, such differential binding could still be identified in the peak-level analysis and high resolution analysis might be considered less interesting. High resolution analysis is perhaps most interesting when the same region is identified as a peak in both conditions but different numbers of binding events are identified between conditions. We further decomposed the predicted binding events based on the number of predicted events in the region in each condition. Table S8 shows that although many regions are occupied in both conditions, the number of predicted binding events can differ significantly. Figure S14 depicts an example of differential occupancy of closely located binding sites in the promoter region of *gltA* gene. Specifically, two binding sites are predicted by dPeak in anaerobic condition while only one of them are identified in aerobic condition. In contrast, MOSAiCS identified the region covering both binding sites as a single peak in both aerobic and anaerobic conditions.

	Anaerobic condition			
Aerobic condition	0	1	2	≥ 3
0	N/A	60	19	1
1	63	198	74	7
2	16	48	235	34
≥ 3	1	3	24	30

Table S8: Cross tabulation of number of binding events for each peak of σ^{70} PET ChIP-Seq data between aerobic and anaerobic conditions.

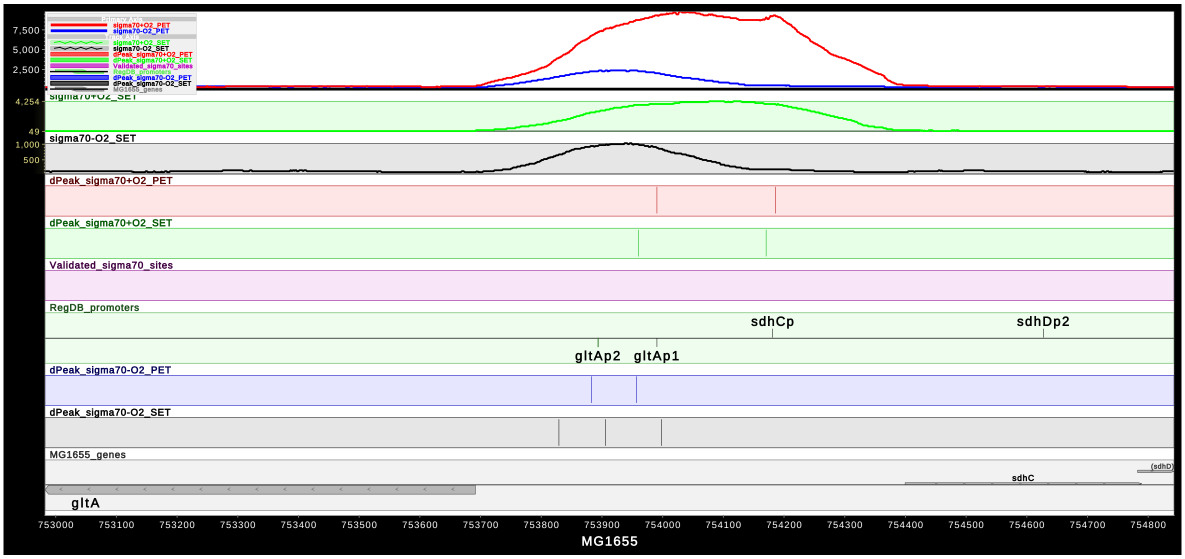

Figure S14: MochiView genome browser [16] screenshots of promoter region of *gltA* gene. Both *gltAp1* and *gltAp2* are identified as binding sites in anaerobic condition using PET ChIP-seq data while only *gltAp1* is identified in aerobic condition.

16 Evaluations of the Algorithms for PET ChIP-Seq Data

To the best of our knowledge, SIPeS is currently the only algorithm specifically designed for supporting PET ChIP-Seq data and has been shown to attain better resolution than a version of MACS that can analyze PET data [17]. We used C implementation version 2.0 of SIPeS from `http://gmdd.shgmo.org/Computational-Biology/ChIP-Seq/download/SIPeS`. In our computational experiments and data analysis, we both used its default parameters and also considered alternative values for the parameters that define the range of the dynamic baseline to construct the signal map. SIPeS constructs signal map by piling up the aligned paired-end reads. [17] observed that SIPeS was able to attain high resolution for binding event identification when used with a wide range of dynamic baseline. Therefore, we investigated the performance of SIPeS when the DNA fragment pileups corresponding to two binding events are above (Figures S15A, B) and within the range of dynamic baseline (Figure S15C) in our computational experiments as described in the main manuscript. We observed that tuning the range of the dynamic baseline is far from trivial. Furthermore, a global value across the whole genome is not likely to perform well. There are also no guidelines or objective ways of configuring such a range.

Figure S15A shows that SIPeS has low sensitivity when two binding events are closely spaced. In this case, the value of the DNA fragment pileups between these two binding events did not belong to the range of dynamic baseline and, as a result, SIPeS identified the whole region as a single peak. Hence, although two binding events reside within this peak, SIPeS reported only a single *summit*. In contrast, Figure S15B shows that, on average, SIPeS identified more than 10 binding events when the distance between two binding events is larger than average library size. For these settings, there were some regions with low DNA fragment pileup within the range of the dynamic baseline between the two binding events. As a result, SIPeS essentially identified all local maxima as binding events and this resulted in low positive predictive value of SIPeS.

When the values of the DNA fragment pileups corresponding to the binding events are within the range of dynamic baseline, SIPeS is able to identify the two binding events (Figure S15C). However, SIPeS also identified all other local maxima as binding events and exhibited significant loss of positive predictive value. We also note that the SIPeS predictions corresponding to true binding events could not be distinguished from others, using the other summary statistics such as p-value or maximum fragment pileup value provided by SIPeS.

Finally, we evaluated SIPeS predictions for the 8 experimentally validated re-

gions of σ^{70} PET ChIP-Seq data. As discussed in the manuscript, these regions harbor a total of 14 experimentally validated σ^{70} binding sites. Figure S15D illustrates that dPeak attains significantly higher resolution compared to SIPeS in these regions (p-value of the paired t-test between dPeak and SIPeS < 0.01). Furthermore, although these regions harbor at most two validated σ^{70} binding sites, SIPeS predicted 2 to 18 binding sites. In summary, SIPeS does not sufficiently leverage PET ChIP-Seq data to provide high resolution for studying protein-DNA interactions. Furthermore, it is also highly sensitive to background noise in ChIP-Seq data and requires parameter tuning. We also note that the analysis of high depth PET ChIP-Seq data, such as that of σ^{70}, using SIPeS requires considering wider ranges of dynamic baseline. This, in turn, increases the computation time significantly. Overall, it seems computationally prohibitive to implement a genome-wide analysis of such data using SIPeS, i.e., analysis of σ^{70} required more than 72 hours on a standard 64 bit machine with Intel Xeon 3.0GHz processor.

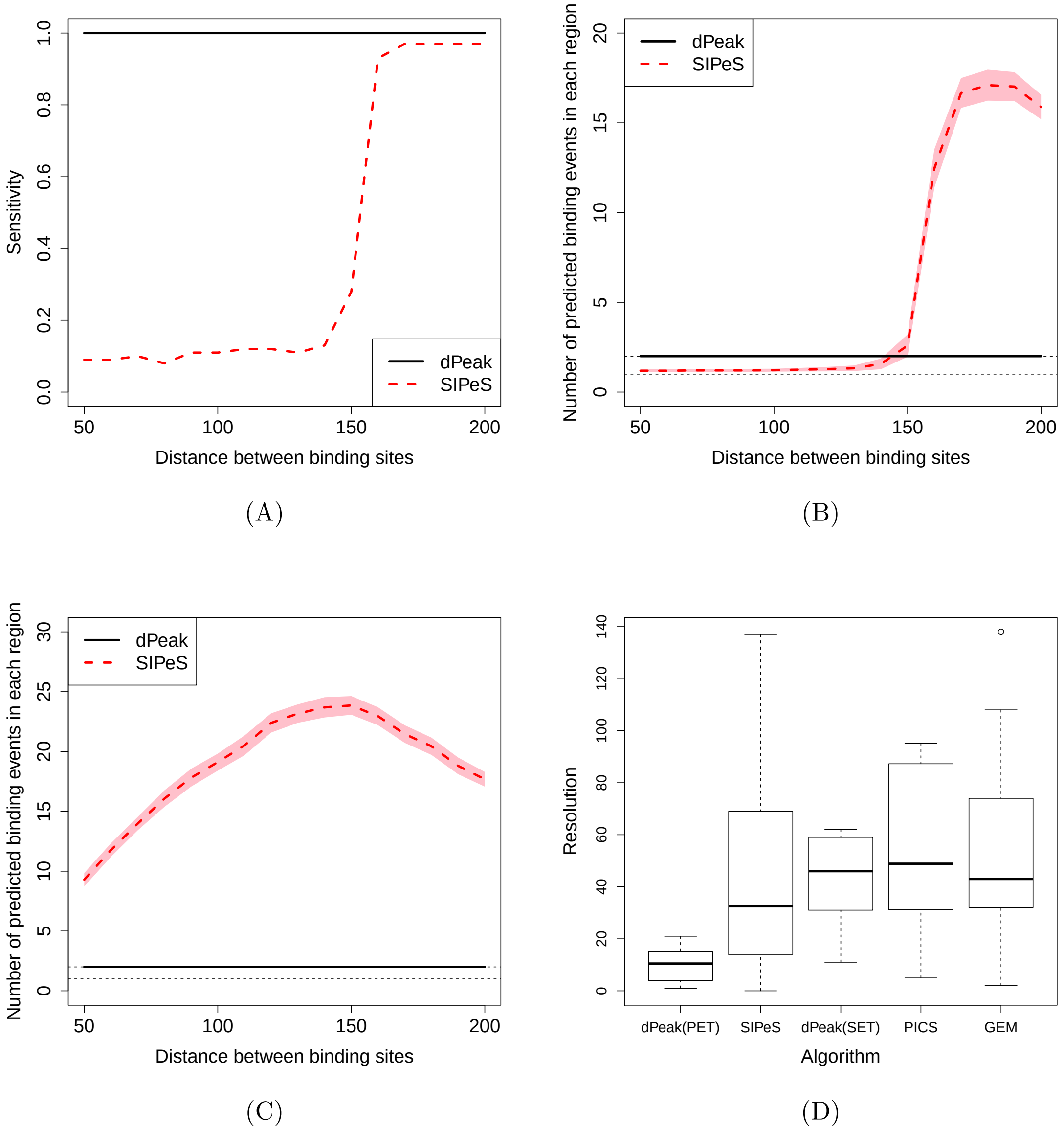

Figure S15: Evaluation of the SIPeS algorithm on PET ChIP-Seq data. Sensitivity and positive predictive value comparisons of SIPeS and dPeak for the computational experiments of PET ChIP-Seq data when DNA fragment pileup corresponding to two binding events is not (A, B) and is (C) within the range of dynamic baseline of SIPeS. (D) Resolutions of predictions for σ^{70} PET and quasi-SET ChIP-Seq data using experimentally validated binding sites as a gold standard.

17 Application of dPeak to a GATA1 SET ChIP-Seq Peak

In this section, we discuss an application of dPeak in eukaryotic genomes using the GATA1 SET ChIP-Seq data from [18]. This dataset has 106,381,508 reads and measures GATA1 occupancy in G1E-ER4 cells after estradiol treatment. GATA1 is known to bind to short consensus sequence WGATAR (W = A or T, R = A or G) [19]. A typical GATA1 ChIP-Seq peak on average harbors 2.32 WGATAR sites in this dataset. Being able to identify which of these are occupied is important for refining consensus sequences and deriving functional roles of about 7 million WGATAR sites in the mouse genome. Figure S16 displays coverage plot of the GATA switch site of the GATA2 locus (-2.8 kb). This region contains four WGATAR motifs separated by 20*bp* to 109*bp*. dPeak predicts that GATA1 factor binds to the second consensus site.

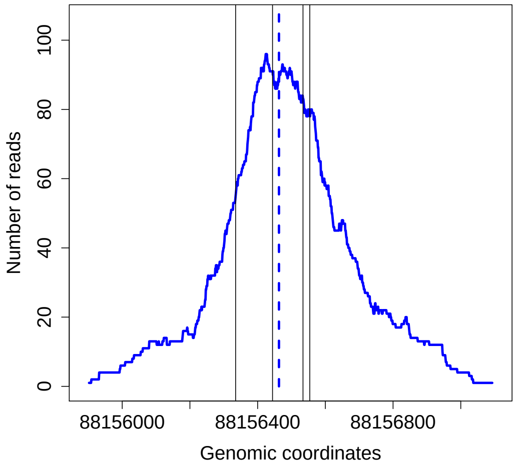

Figure S16: Coverage plot and dPeak prediction for the GATA switch site of the GATA2 locus. Blue curve and blue dotted vertical line indicate the GATA1 SET ChIP-Seq data from [18] and the prediction using the dPeak algorithm, respectively. Black solid vertical lines indicate positions of the GATA1 consensus sequences, [AT]GATA[AG].

18 Evaluations on Human SET ChIP-Seq Data

We evaluated the performance of dPeak on human SET ChIP-Seq data that GPS and PICS were optimized for. We considered GABPA SET ChIP-Seq data in GM12878 cell line from the ENCODE database. We identified 2,469 candidate regions using MOSAiCS (FDR = 1e-20) and these candidate regions were explicitly provided to the GPS and GEM algorithms as candidate regions. Candidate regions for PICS were identified using the function `segmentReads()` in the `PICS` R package (default parameters). Default tuning parameters were used during model fitting for all the methods.

In the case of a sequence-specific factor with well-conserved motif such as the GABPA factor, we observed that dPeak prediction can be further improved in a straightforward way by incorporating sequence information. Specifically, after identifying initial dPeak predictions, we identified a *de novo* motif using MEME [20] and detected positions of these consensus sequences using FIMO [21]. Then, we updated the dPeak predictions if the GABPA consensus sequences were found within the 50*bp* window around initial dPeak predictions. We call these dPeak predictions that integrate sequence information as 'dPeak2'.

Figure S17 shows resolution comparison on the GABPA-GM12878 dataset. The resolution is defined as the absolute distance to the nearest predicted consensus site, where the prediction utilizes the independent position weight matrix from JASPAR [22]. The results indicate that dPeak performs comparable to GPS (median resolution = 18*bp* and 19*bp* for dPeak and GPS, respectively) and they both significantly outperform PICS (median resolution = 30*bp*). Moreover, dPeak2 performs comparable to GEM and identifies the GABPA binding sites with high accuracy.

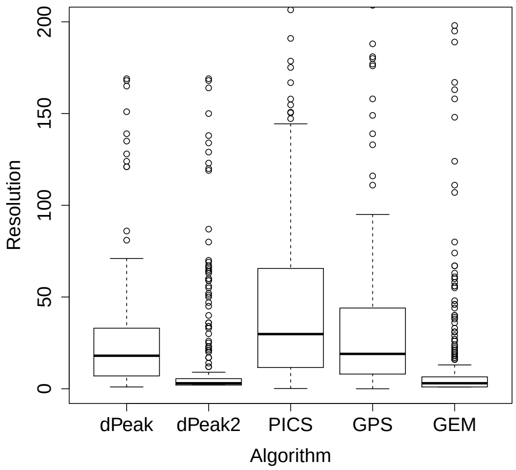

Figure S17: Resolutions of predictions for ENCODE GABPA-GM12878 SET ChIP-Seq Data, using positions of GABPA consensus sequences as identified by the JASPAR position weight matrix scan as a gold standard.

19 Comparison of dPeak using SET ChIP-Seq with ChIP-exo

ChIP-exo [23] is a modified ChIP-Seq protocol that aims to experimentally identify binding sites at high resolution by employing exonuclease. ChIP-exo protocol is more laborious compared to ChIP-Seq and there are not many available ChIP-exo datasets yet. Despite these limitations, we investigated how ChIP-Seq analysis with dPeak compared to ChIP-exo analysis for identifying binding sites in high resolution. We evaluated ChIP-exo data measuring binding of CTCF factor in human HeLa-S3 cell line (downloaded from SRA with accession number SRA044886). Although [23] did not generate ChIP-Seq data in parallel to this ChIP-exo data, we were able to utilize SET ChIP-Seq data for CTCF factor in human HeLa-S3 cell line from the ENCODE project (Crawford Lab, Duke University). For both ChIP-exo and ChIP-Seq data, all the available replicates were combined.

In order to evaluate the performance of ChIP-exo data, we utilized predictions provided in [23]. These predictions were generated using a combination of an automated tool for analyzing ChIP-exo data in a strand-specific manner and a set of manually curated rules by inspection of the data. For comparison, we also generated predictions of dPeak, GPS, and GEM for CTCF ChIP-exo and SET ChIP-Seq data. We did not consider PICS because it is not tailored for the ChIP-exo data analysis. We also generated dPeak2 predictions by utilizing sequence information using the same procedure as described in Section 18. We utilizes the CTCF position weight matrix from JASPAR [22], as a gold standard.

Figure S18 shows proportion of CTCF consensus sequences identified by each method at given spatial resolution. The results indicate that dPeak and dPeak2 using ChIP-exo data shows spatial resolution comparable to or better than predictions of [23], GPS, and GEM, which implies that dPeak can readily be utilized in ChIP-exo data analysis. It also shows that predictions using CTCF ChIP-Seq data provide significantly higher spatial resolution compared to predictions using CTCF ChIP-exo data.

References

[1] Zhang Y, Liu T, Meyer CA, Eeckhoute J, Johnson DS, et al. (2008) Model-based analysis of ChIP-Seq (MACS). Genome Biol 9: R137.

[2] Kuan PF, Chung D, Pan G, Thomson JA, Stewart R, et al. (2011) A statistical framework for the analysis of ChIP-Seq data. J Am Stat Assoc 106: 891–903.

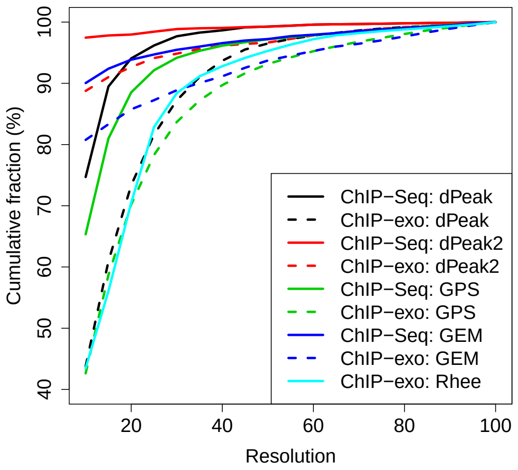

Figure S18: Comparison of cumulative fraction as a function of spatial resolution, for ChIP-exo data and SET ChIP-Seq data of CTCF factor in human HeLa-S3 cell line. Cumulative fraction is defined as proportion of CTCF consensus sequences identified by each method at a given spatial resolution.

[3] Dempster AP, Laird NM, Rubin DB (1977) Maximum likelihood from incomplete data via the EM algorithm. J R Stat Soc, Series B 39: 1–38.

[4] Meng XL, Rubin DB (1993) Maximum likelihood estimation via the ECM algorithm: a general framework. Biometrika 80: 267–278.

[5] McLachlan G, Peel D (2000) Finite Mixture Models. Wiley, New York.

[6] McLachlan G, Krishnan T (2008) The EM Algorithm and Extensions. Wiley, New York.

[7] Celeux G, Diebolt J (1985) The SEM algorithm: a probabilistic teacher algorithm derived from the EM algorithm for the mixture problem. Comput Stat Q 2: 73–82.

[8] Crawford S (1994) An application of the Laplace method to finite mixture distributions. J Am Stat Assoc 89: 259–267.

[9] Schwarz G (1978) Estimating the dimension of a model. Ann Stat 6: 461–464.

[10] Fraley C, Raftery A (1998) How many clusters? Which clustering method? Answers via model-based cluster analysis. Comput J 41: 578–588.

[11] Fraley C, Raftery AE (2002) Model-based clustering, discriminant analysis, and density estimation. J Am Stat Assoc 97: 611–631.

[12] Gama-Castro S, Salgado H, Peralta-Gil M, Santos-Zavaleta A, Muniz-Rascado L, et al. (2011) RegulonDB version 7.0: transcriptional regulation of *Escherichia coli* K-12 integrated within genetic sensory response units (Gensor Units). Nucleic Acids Res 39: D98–D105.

[13] Zhang X, Robertson G, Krzywinski M, Ning K, Droit A, et al. (2011) PICS: probabilistic inference for ChIP-seq. Biometrics 67: 151–163.

[14] Guo Y, Papachristoudis G, Altshuler RC, Gerber GK, Jaakkola TS, et al. (2010) Discovering homotypic binding events at high spatial resolution. Bioinformatics 26: 3028–3034.

[15] Guo Y, Mahony S, Gifford DK (2012) High resolution genome wide binding event finding and motif discovery reveals transcription factor spatial binding constraints. PLoS Comput Biol 8: e1002638.

[16] Homann OR, Johnson AD (2010) MochiView: versatile software for genome browsing and DNA motif analysis. BMC Biol 8: 49.

[17] Wang C, Xu J, Zhang D, Wilson Z, Zhang D (2010) An effective approach for identification of *in vivo* protein-DNA binding sites from paired-end ChIP-Seq data. BMC Bioinformatics 11: 81.

[18] Wu W, Cheng Y, Keller CA, Ernst J, Kumar SA, et al. (2011) Dynamics of the epigenetic landscape during erythroid differentiation after GATA1 restoration. Genome Res 21: 1659–1671.

[19] Bresnick EH, Lee HY, Fujiwara T, Johnson KD, Keleş S (2010) GATA switches as developmental drivers. J Biol Chem 285: 31087-31093.

[20] Bailey TL, Elkan C (1994) Fitting a mixture model by expectation maximization to discover motifs in biopolymers. ISMB94 : 28–36.

[21] Grant CE, Bailey TL, Noble WS (2011) FIMO: Scanning for occurrences of a given motif. Bioinformatics 27: 1017-1018.

[22] Bryne JC, Valen E, Tang MH, Marstrand T, Winther O, et al. (2008) JASPAR, the open access database of transcription factor-binding profiles: new content and tools in the 2008 update. Nucleic Acids Res 36: D102–D106.

[23] Rhee HS, Pugh BF (2011) Comprehensive genome-wide protein-DNA interactions detected at single-nucleotide resolution. Cell 147: 1408-1419.
